# Oxygen transfer reaction of haloalkyl amides catalyzed by phenylboronic acid

**DOI:** 10.1038/s42004-023-00824-6

**Published:** 2023-02-10

**Authors:** Abhijit Sen, Atsuya Muranaka, Aya Ohno, Yoichi M. A. Yamada

**Affiliations:** RIKEN Center for Sustainable Resource Science, Wako, Saitama, 351-0198 Japan

**Keywords:** Synthetic chemistry methodology, Organocatalysis, Catalytic mechanisms, Reaction mechanisms

## Abstract

Nitrile derivatives are important building blocks in organic synthesis. Herein, we report the serendipitous discovery of an oxygen transfer reaction that produces hydroxyalkyl nitriles from the sequential dehydration and hydrolysis of haloalkyl amides. Product yields of up to 91% were achieved, and the phenylboronic acid was recovered as triphenylboroxine. The triphenylboroxine was reused as a catalyst without any loss of catalytic activity. A probable catalytic pathway was proposed based on control experiments and DFT calculations.

## Abstract (web summary)

Amide dehydration is an important reaction to access organonitrile compounds harboring high synthetic value, however, the reaction relies on harsh dehydrating reagents or transition metals. Here, the authors develop an oxygen transfer reaction to generate hydroxyalkyl nitriles from sequential dehydration and hydrolysis of haloalkyl amides catalyzed by phenylboronic acid.

## Introduction

**Fig. 1:**
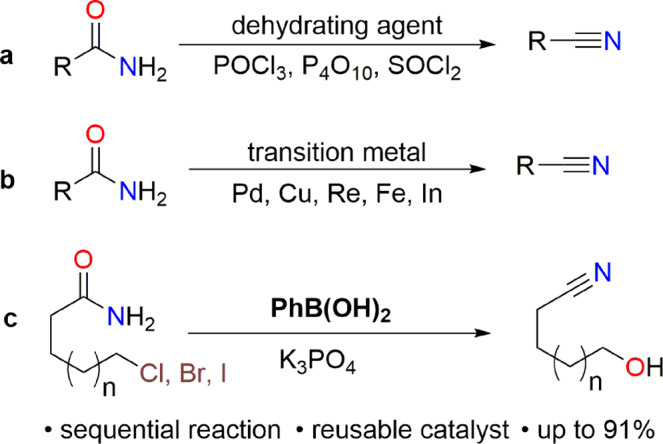
Representative examples of nitrile derivative synthesis from amides. **a** Dehydration of primary amide using dehydrating agents. **b** Transition metal-catalyzed dehydration of amide. **c** Oxygen transfer reaction (this study).

Organonitrile compounds are important to synthesis in medicinal, biological, and materials chemistry^1–5^ because of the very unique reactivity and activating ability of the nitrile group^6–8^. There are several methods for the synthesis of nitrile derivatives^9–13^, which are mainly based on the transition-metal-catalyzed reaction of aryl/alkyl halides or alcohols^14–21^. In addition, amide dehydration is an important method for the construction of the nitrile moiety^22–24^. Previously, the dehydration of a primary amide was conducted using harsh and acidic dehydrating reagents such as POCl_3_^22^, P_4_O_10_^23^, and SOCl_2_^24^ (Fig. 1a). Recently, there has been significant interest in the dehydration of amides^25–33^ catalyzed by transition metals such as Pd^26–29^, Re^30^, Fe^32^, In^31^ and Cu^33^ (Fig. 1b). On the other hand, the hydrolysis of alkyl halides to generate the corresponding alcohols is a textbook reaction^34–36^. Hydroxyl and nitrile groups are important because both of these functionalities can be found in several biologically active molecules and naturally occurring substrates^5,37–41^.

A domino conversion of amide to nitrile and alkyl halide to the corresponding alcohol would be a very straightforward strategy for the construction of hydroxyalkyl nitriles (Fig. 1c). Herein, we report oxygen transfer reaction of haloalkyl amides affording hydroxyalkyl nitriles using a metal-free catalyst, phenylboronic acid, which can be reused without loss of catalytic activity.

## Results and discussion

### Reaction discovery

**Fig. 2:**
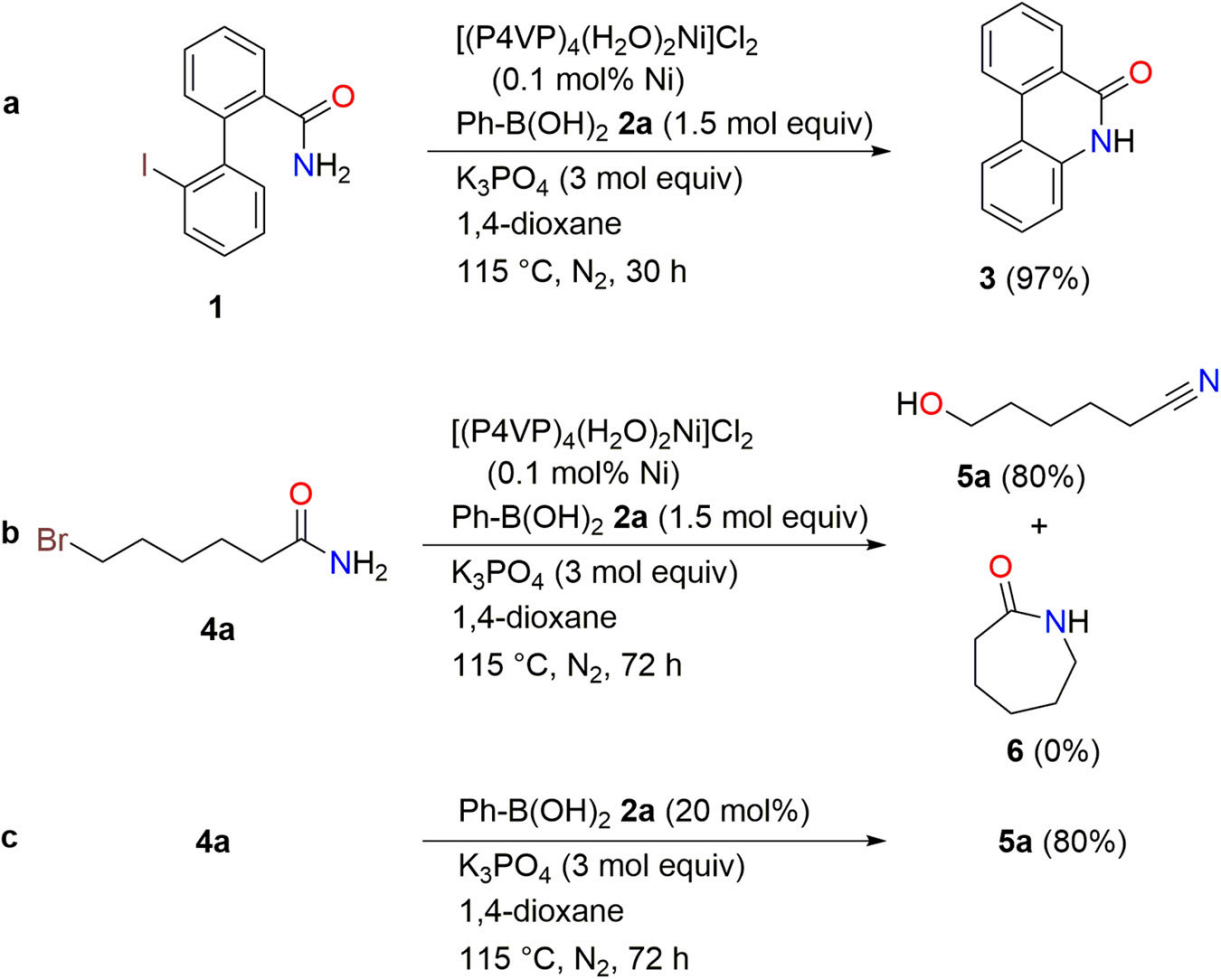
Serendipitous reaction discovery. **a** Our previous report of nickel-catalyzed lactamization. **b** Discovery of oxygen transfer reaction. **c** Phenylboronic acid-catalyzed oxygen transfer reaction.

We previously reported the formation of lactams, phenanthridinone (**3**) by intramolecular amidation of aryl halides and amides using a polymeric nickel catalyst and phenylboronic acid (Fig. 2a)^42^. However, the reaction of 6-bromohexanamide (**4a**) surprisingly produced 6-hydroxyhexanenitrile (**5a**) in 80% yield instead of lactam **6** (Fig. 2b). Eventually, phenylboronic acid (**2a**) became the catalyst^43–49^ in this transformation, and the oxygen transfer reaction of **4a** with 20 mol% of **2a** and 3 molar equiv of potassium phosphate gave **5a** in 80% yield (Fig. 2c and Table 1, entry 1).

**Table 1:** Deviation from standard reaction conditions^a^.


Entry	Deviation from standard conditions	Yield (%)
1	None	80
2	2-phenyl-1,3,2-dioxaborinane (**7**) instead of **2a**	72
3	10 mol% of **2a** instead of 20 mol%	60
4	Without **2a**	0
5	Toluene instead of 1,4-dioxane	72
6	KO*t*Bu instead of K_3_PO_4_	0

^a^The reaction of **4a** (1 molar equiv), **2a** (20 mol%), and K_3_PO_4_ (3 molar equiv) was conducted at 115 °C for 72 h.

The effects of deviation from the standard reaction conditions are shown in Tables 1 and S1 (Supporting Information). The use of 20 mol% of 2-phenyl-1,3,2-dioxaborinane (**7**) as the catalyst gave 72% of **5a** (entry 2), which was similar as the yield of the reference reaction (80%, entry 1). The reaction afforded 60% of 5a when the amount of **2a** was reduced from 20 to 10 mol% (entry 3). In the absence of **2a**, no reaction occurred, confirming that this compound is the catalyst in this transformation (entry 4). The reaction in toluene as the solvent afforded **5a** in 72% yield (entry 5). When a stronger base, potassium tert-butoxide (KO*t*Bu), was used, the desired product **5a** was not obtained (entry 6), and the simple β-elimination of the terminal alkyl bromide moiety occurred instead (see Supporting Information for more details).

### Substrate scope

**Fig. 3:**
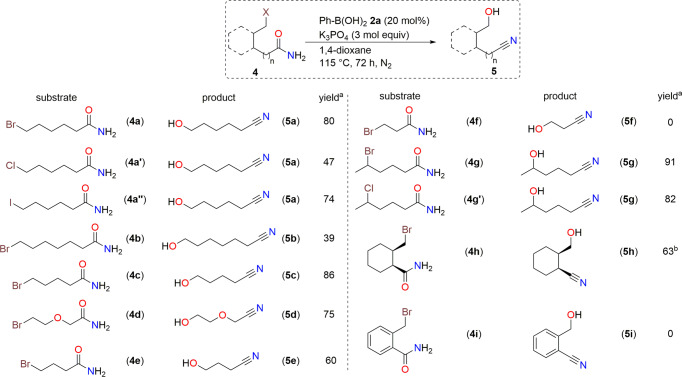
Substrate scope of amide dehydration and alkyl halide hydrolysis. Reaction conditions: **4** (1 mol equiv), **2a** (20 mol%), and K_3_PO_4_ (3 mol equiv) at 115 °C for 72 h. ^a^isolated yield. ^b^yield was determined after tosylation.

With the optimized reaction conditions in hand, we examined the substrate scope of this reaction (Fig. 3). The reaction of **4a** produced **5a** in 80% yield. The reactions of 6-chlorohexanamide (**4a’**) and 6-iodohexanamide (**4a”**) also afforded **5a** in 47 and 74% yield, respectively. The reaction of 7-bromoheptanamide furnished 7-hydroxyheptanenitrile (**5b**) in 39% yield. The conversion of 5-bromopentanamide (**4c**) afforded 5-hydroxypentanenitrile (**5c**) in higher yield (86%). The reaction of the oxygen-tethered 2-(2-bromoethoxy)acetamide (**4d**) afforded **5d** in 75% yield. Similarly, 4-bromobutanamide (**4e**) provided **5e** in 60% yield. Unfortunately, the reaction of 3-bromopropanamide (**4f**) did not proceed. Here acrylamide was observed as the major product (see Supporting Information for more details). The reaction of a secondary alkyl bromide, 5-bromohexanamide (**4g**), showed the best result, providing **5g** in 91% yield. Moreover, a secondary alkyl chloride, 5-chlorohexanamide (**4g’**), was also converted to **5g** in 82% yield. The reaction of the cyclic compound, 2-(bromomethyl)cyclohexane-1-carboxamide (**4h**), proceeded to afford **5h** in 63% yield. The yield of **5h** was determined after tosylation (see Supporting Information for more details). The reaction of an aromatic amide (**4i**) was unsuccessful where lactam was obtained as a major product.

### Catalyst reusability

**Fig. 4:**
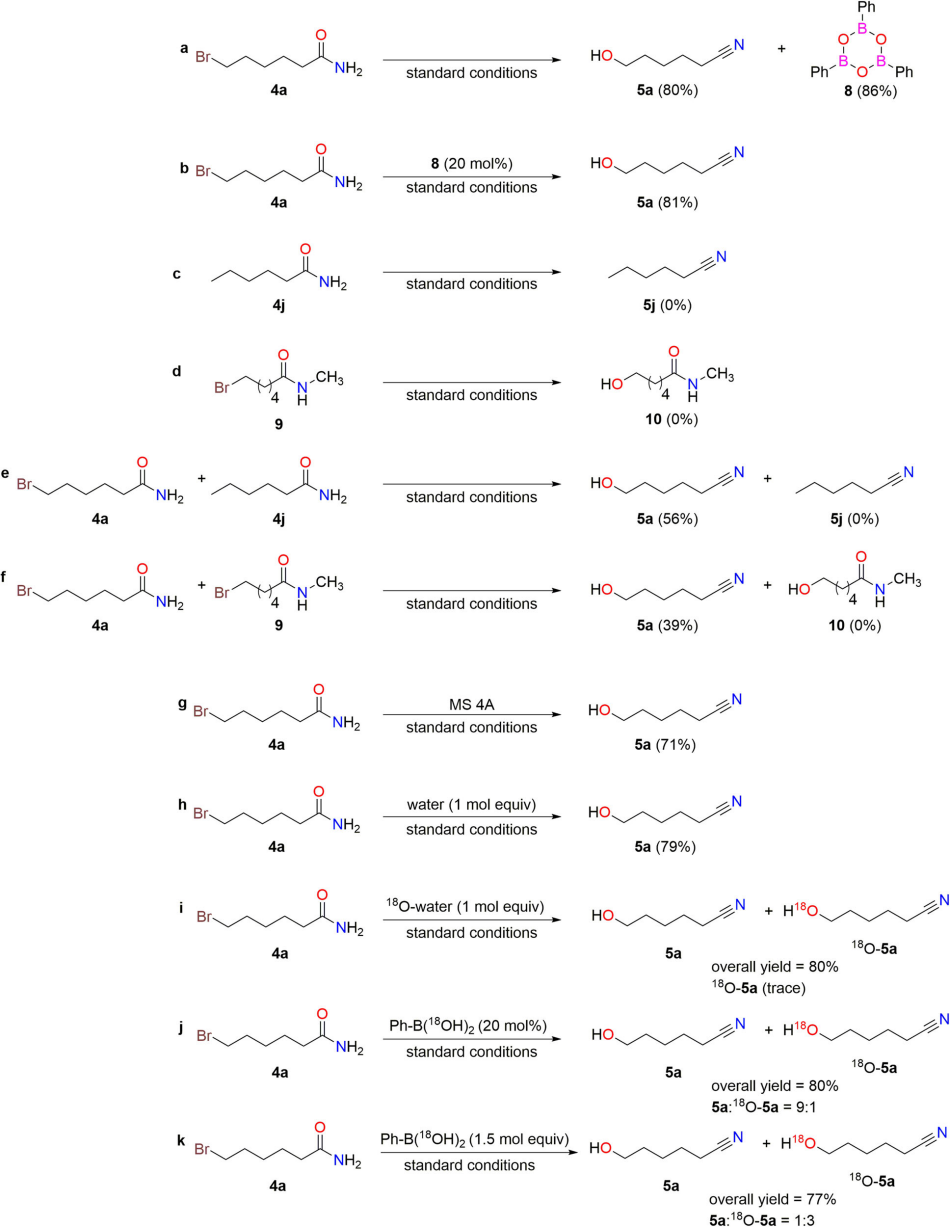
Catalyst reusability and control experiments. **a** Catalyst recovery. **b** Reusability of recovered catalyst **8**. **c** Dehydration of amide without halogen tethering. **d** Hydrolysis of secondary amide under standard reaction conditions. **e** Intermolecular competition with/without halogen tethering. **f** Intermolecular competition between primary and secondary amide. **g** The effect of molecular sieves. **h** the effect of water. **i** The effect of ^18^O-water. **j** The effect of 20 mol% of Ph-B(^18^OH)_2_. **k** the effect of 1.5 molar equiv of Ph-B(^18^OH)_2_.

The reusability of the catalyst was then investigated (Fig. 4a, b). After the reaction, the catalyst was recovered as a cyclic trimer (phenylboroxine, **8**) in 86% yield through column chromatographic separation (Fig. 4a). When the recovered **8** was used as the catalyst, the reaction proceeded efficiently to provide 81% yield of **5a** (Fig. 4b).

### Control experiments

The reaction of **4j** (a halogen-free substrate, Fig. 4c) and **9** (a secondary amide with no possibility of water generation, Fig. 4d) were not converted to the corresponding nitriles and alcohols, respectively. The reaction of **4a** proceeded to afford **5a** in the presence of **4j** (Fig. 4e) and **9** (Fig. 4f) whereas the corresponding nitrile (**5j**) and alcohol (**10**) were not observed. The yield of **5a** was dropped slightly to 71% when molecular sieves (4A) were added to the reaction mixture as dehydrating agents (Fig. 4g). Contrarily, the yield of **5a** was neither increased nor decreased in presence of one mol equiv of water (Fig. 4h). The addition of ^18^O-water produced a trace amount of ^18^O-**5a** product (Fig. 4i) which suggests water is not directly involved in this reaction but it could take part via the equilibrium between phenylboronic acid and its corresponding anhydride^50^. Contrarily, the amount of ^18^O-**5a** was increased significantly (**5a**:^18^O-**5a** = 9:1) when ^18^O-**2a** (^16^O_2_:^16^O^18^O:^18^O_2_ = 14:23:64)^51^ was used as catalyst (Fig. 4j).In addition, ^18^O-**5a** was observed as the main product(**5a**:^18^O-**5a** = 1:3) when 150 mol% (1.5 molar equiv) of ^18^O-**2a** was used as additive (Fig. 4k). This increase of ^18^O in the product suggest the oxygen transfer proceeded via the phenylboronic acid.

### Plausible catalytic pathway

**Fig. 5:**
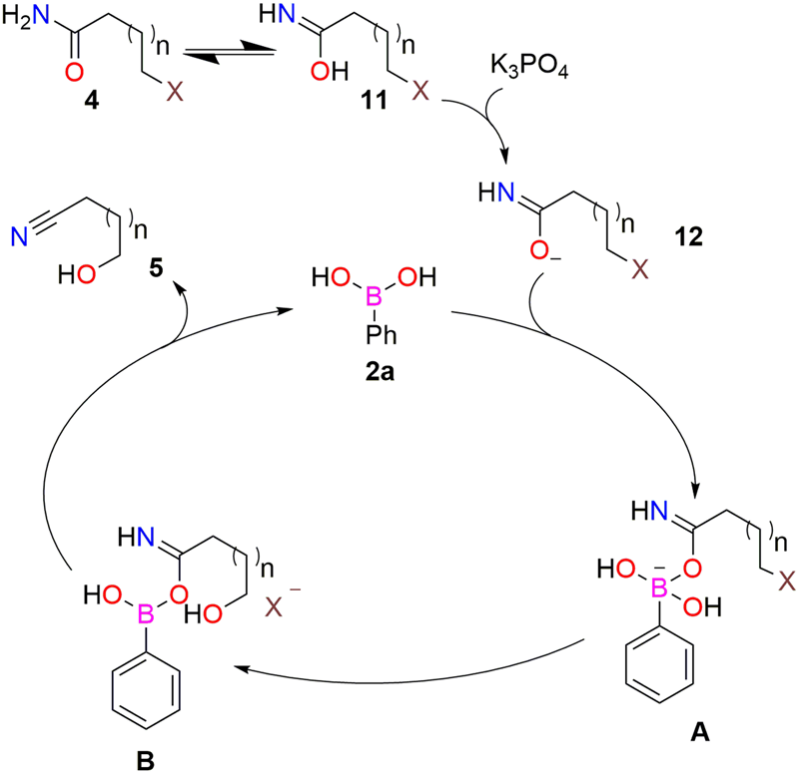
Plausible catalytic pathway. Initial deprotonation of amide in the presence of a base lowers the activation energy to form A.

A plausible catalytic pathway is proposed in Fig. 5. Initially, the amide substrates (**4**) present in a equilibrium with the corresponding imidic acid intermediate (**11**). Next, the base (K_3_PO_4_) deprotonate the intermediate **11** to form the intermediate **12**. Next, intermediate **12** reacts with phenylboronic acid (**2a**) to form intermediate **A**. The hydroxyl group attached with boron acts as a nucleophile and substitutes the halide ion via nucleophilic substitutuion reaction to form intermediate **B**. Finally, a carbon-nitrogen triple (nitrile) bond formation regenerates the phenylboronic acid (**2a**) and affoard the product (**5**). Phenylboroxine (**8**) might generate after the end of the catalytic cycle.

### DFT studies

**Fig. 6:**
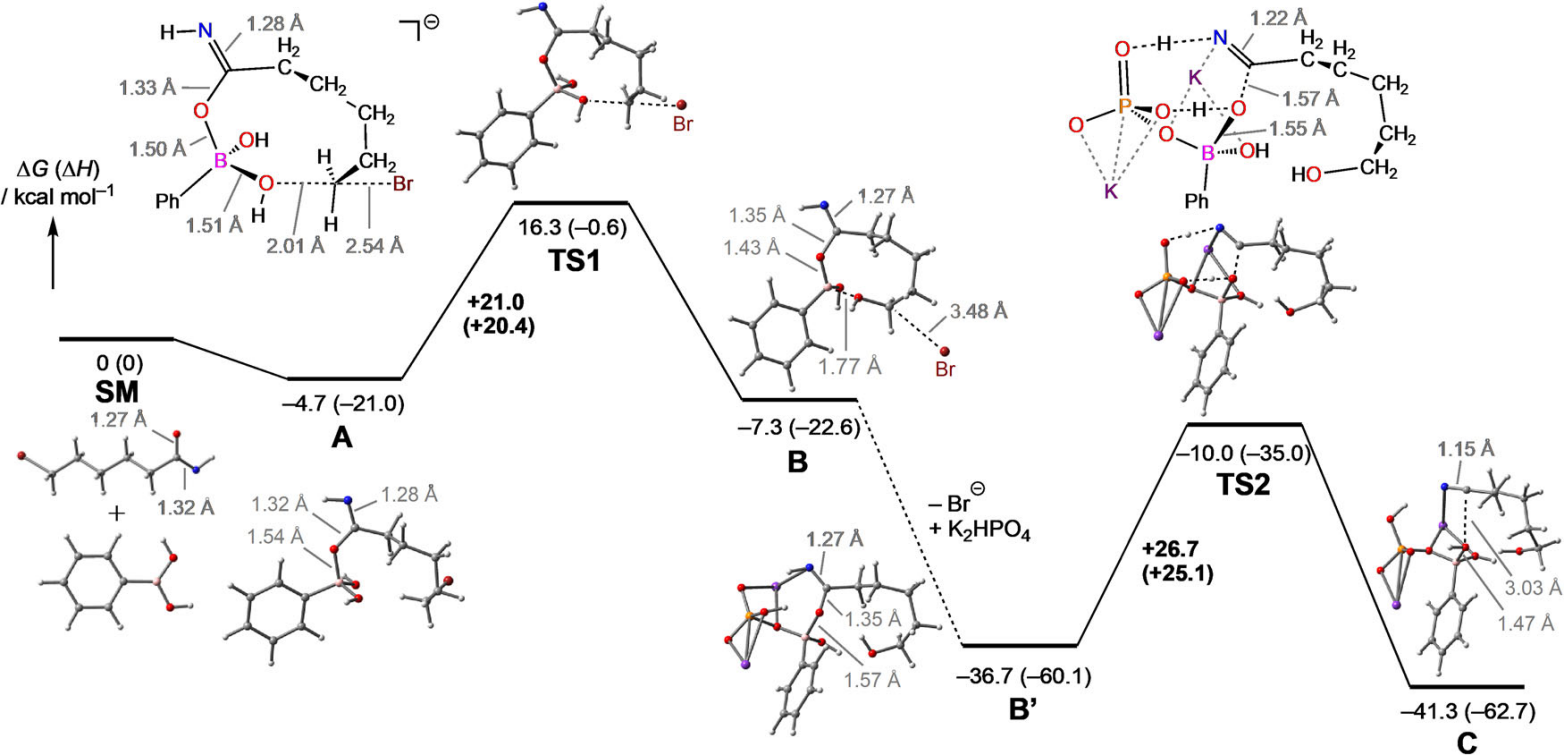
Calculated reaction energy diagram for the oxygen transfer reaction (M06-2X/6-311 + G(d, p)). The C–O bond cleavage and nitrile formation procceds through a single transition state (**TS2**).

To support our proposed reaction mechanism, we performed DFT calculations (see the Supporting Information and Supplementary Figs. 1 and 2). As shown in Fig. 6, our calculations suggested that the reaction proceeds through a two-step mechanism. First, a deprotonated amide substrate interacts with boronic acid **2a** to form a tetrahedral boron intermediate **A**. An intramolecular nucleophilic substitution of the bromine with one OH group then occurs via the transition state **TS1**, which results in the intermediate **B**. The transition state was calculated to be 21.0 kcal/mol higher in energy compared to intermediate **A**. The second step is the cleavage of the C–O bond and formation of carbon-nitrogen triple bond. We found that K_2_HPO_4_ plays an essential role in the second step. One oxygen atom of K_2_HPO_4_ is coordinated to the boron atom in intermediate **B’** where NH and POH protons are directed towards the PO and CO oxygen atoms, respectively. The NH and POH protons simultaneously transfer through a single transition state (**TS2**) to give the corresponding hydroxyalkyl nitrile (intermediate **C**). The activation energy for **TS2** (26.7 kcal/mol) was slightly higher than that of **TS1**.

## Conclusion

In summary, we found an oxygen transfer reaction during the sequential dehydration of primary alkyl amides and hydrolysis of alkyl halides to afford hydroxyalkyl nitriles, which was catalyzed by phenylboronic acid. In this reaction, a wide variety of substrates were tolerated and the catalyst could be recovered and reused. The reaction pathway for this unique transformation was proposed based on DFT calculations and control experiments. Complete mechanistic studies are now ongoing in our laboratory.

## Methods

General procedure for the synthesis of hydroxynitrile derivatives. A mixture of phenylboronic acid **2a** (20 mol%, 24.2 mg), an amide **4** (1 mol equiv, 1 mmol), and K_3_PO_4_ (3 mol equiv, 636 mg) was added to a reaction tube (see supplementary methods). The reaction tube was degassed under vacuum and refilled with N_2_ under standard Schlenk techniques (3 times). To the reaction mixture, 1,4-dioxane was added (2 mL). The reaction tube was sealed with a screw cap and teflon and then stirred at 115 °C under nitrogen for 72 h. Finally, the reaction was quenched with 1 N HCl, and the reaction mixture was extracted with EtOAc. The EtOAc layer was collected and dried over Na_2_SO_4_. The solvent was evaporated under vacuum, and the resulting crude mixture was purified by column chromatography (hexane/ethyl acetate) to give product **5**.

## Supplementary information

Supplementary Information

Description of Additional Supplementary Files

Supplementary Data 1

Supplementary Data 2

## Data Availability

All the data created for this studies (compound characterization, ^1^H-NMR, ^13^C-NMR, HRMS, melting point) are present in supporting information file (PDF), the cartesian coordinates for the DFT studies are shown in Supplementary Data 1 File (PDF), the NMR and GC spectras are shown in Supplementary Data 2 File (PDF).
