# Publisher Correction: Oxygen transfer reaction of haloalkyl amides catalyzed by phenylboronic acid

**DOI:** 10.1038/s42004-023-00842-4

**Published:** 2023-02-22

**Authors:** Abhijit Sen, Atsuya Muranaka, Aya Ohno, Yoichi M. A. Yamada

**Affiliations:** RIKEN Center for Sustainable Resource Science, Wako, Saitama, 351-0198 Japan

**Keywords:** Synthetic chemistry methodology, Organocatalysis, Catalytic mechanisms, Reaction mechanisms

Correction to: *Communications Chemistry* 10.1038/s42004-023-00824-6, published online 10 February 2023.

The original version of this Article contained errors in the description of the plausible catalytic pathway, whereby intermediates **11** and **12** were incorrectly designated as **9** and **10**, respectively, in the second, third and fourth sentences of this section.

In addition, bold font was missing from all compound numbers in the second paragraph of the Reaction discovery section, the caption of Figure 4, and the Methods section.

These errors have now been corrected in both the PDF and HTML versions of the Article.

