# Supplementary material for: Oxygen transfer reaction of haloalkyl amides catalyzed by phenylboronic acid
Source: Commun Chem. 2023 Feb 10;6:29. doi: 10.1038/s42004-023-00824-6 (PMC9918490; doi:10.1038/s42004-023-00824-6)
Supplement: Supplementary file 1 — Supplementary Information [file 42004_2023_824_MOESM1_ESM.pdf]

Supporting Information

Oxygen Transfer Reaction of Haloalkyl Amides Catalyzed by Phenylboronic Acid

Abhijit Sen, Atsuya Muranaka, Aya Ohno, and Yoichi M. A. Yamada*

RIKEN Center for Sustainable Resource Science, Wako, Saitama 351-0198, Japan

ymayamada@riken.jp

Contents

1. General S2
2. Preparation of Starting Materials S2
3. Optimization of Different Boron Source S4
4. Catalysis
   General Procedure for Metal Free Dehydration of Amide S5
   Characterization of Products S5
5. DFT Studies for Probable Reaction Mechanism S7
6. List of Side Reaction Products S9
7. Supplementary References S10

Supplementary Methods

1. General

All chemicals and solvents were used as received without further purification unless otherwise mentioned. ^{1}H-NMR (500 MHz), and ^{13}C-NMR (125 MHz) spectra were measured with a JEOL JNM ECA-500 spectrometer at 25 °C. Chemical shifts (δ) are expressed relative to the resonances of the residual non-deuterated solvent for ^{1}H[$CDCl_3$]: ^{1}H (δ) = 7.26 ppm, acetone-d_6: ^{1}H (δ) = 2.05 ppm] and ^{13}C [$CDCl_3$: ^{13}C (δ) = 78.0 ppm, acetone-d_6: ^{13}C = 29.8 and 206.3 ppm]. Absolute values of the coupling constants are given in Hertz (Hz), regardless of their sign. Multiplicities are abbreviated as singlet (s), doublet (d), doublet of doublets (dd), triplet (t), quartet (q), quintet (quint), multiplet (m), and broad (br).
TLC analysis was performed on Merck silica gel 60 F254. Flash column chromatography was carried out using Wakogel silica C-200 (particle size: 75-150 μm). A series of combinations of ethyl acetate and hexane was used as an eluent. ESI-MS mass was determined by using micrOTOFQIII instrument.
The Starting material **4a**[1], **4b**[2], **4c**[3], **4e**[4], **4f**[5], **4h**[6], **9**[7] and ^{18}O-**2a**[8] are prepared by following the literature procedure. The starting materials **4i**, **4j**, **2a** and **7** were purchased from commercial sources and used without further purifications.

2. Preparation of Starting Materials

2.1. General Procedure 1:

A mixture of caprolactone (2 g), 48% HBr (20 mL), and concentrated H_2SO_4 (2.5 mL) was heated under reflux until the completion of the reaction. After cooling to room temperature, 100 mL of water was added to the reaction mixture. The reaction mixture was extracted several times with diethyl ether. The combined organic layer was dried over anhydrous Na_2SO_4 and evaporated in a vacuum. A pale-yellow liquid was obtained as a product. This product was used directly to the next step without further purification. The yellow liquid, **S2** was stirred overnight (15 h) together with 1.5 mL of oxalyl chloride in dichloromethane solvent. The volatiles were removed under reduced pressure and the residue was added drop by drop to 10 mL of ammonium hydroxide solution (28% m/m) with vigorous stirring while keeping the temperature below 10 °C. The solid amide was filtered, washed with ice water, and dried under a vacuum. The desired amide was further purified by recrystallization from hexane/ethyl acetate solvent.

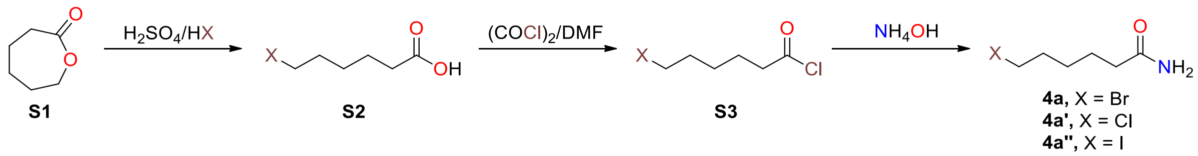

6-bromohexanamide (4a, Ref. 1)

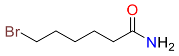

White solid (overall 72% yield, 2.4 g). ^{1}H-NMR (500 MHz, $CDCl_3$) δ 5.47 (brs, 2H), 3.42 (t, J = 5.7 Hz, 2H), 2.22-2.27 (m, 2H), 1.77-1.96 (m, 2H), 1.57-1.74 (m, 2H), 1.34-1.52 (m, 2H). ^{13}C{^{1}H}-NMR (125 MHz, $CDCl_3$) δ 175.6, 35.6, 33.7, 32.5, 27.8, 24.6.

6-chlorohexanamide (4a')

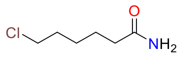

White solid (overall 53% yield, 1.4 g). m. p. 97-98 °C; ^{1}H-1H-NMR (500 MHz, Methanol-d$_4$) δ 3.54 (t, *J* = 6.6 Hz, 2H), 2.20 (t, *J* = 7.4 Hz, 2H), 1.76 (dt, *J* = 21.2, 6.6 Hz, 2H), 1.59-1.65 (m, 2H), 1.43-1.49 (m, 2H). ^{13}C{^{1}H}-NMR (125 MHz, Methano-d$_4$) δ 177.6, 44.2, 34.9, 32.1, 26.2, 24.8. HRMS (ESI): Cald for $C_6H_{12}ClNO$: m/z 150.0680 ([M+H]$^+$), found: m/z 150.0677.

6-iodohexanamide (4a")

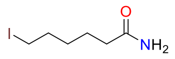

White solid (overall 75% yield, 3g). m.p. 97-98 °C; ^{1}H-NMR (500 MHz, Methanol-d$_4$) δ 3.22 (t, *J* = 6.9 Hz, 2H), 2.19 (t, *J* = 7.4 Hz, 2H), 1.80 (td, *J* = 14.6, 7.1 Hz, 2H), 1.58-1.64 (m, 2H), 1.38-1.46 (m, 2H). ^{13}C{^{1}H}-NMR (125MHz, CDCl$_3$) δ 175.4, 35.6, 33.2, 30.1, 24.4, 6.7. HRMS (ESI): Cald for $C_6H_{12}INO$: m/z 242.0036 ([M+H]$^+$), found: m/z 242.0036.

2.2. Synthesis of 2-(2-bromoethoxy)acetamide, 4d:

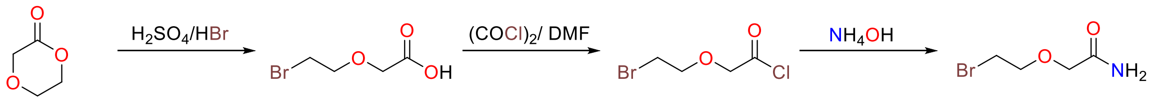

S4 **S5** **S6** **4d**

A mixture of dioxanone (2 g), 48% HBr (20 mL), and concentrated H_2SO_4 (2.5 mL) was heated under reflux until the completion of the reaction. After cooling to room temperature, 100 mL of water was added to the reaction mixture. The reaction mixture was extracted several times with diethyl ether. The combined organic layer was dried over anhydrous Na_2SO_4 and evaporated in a vacuum. A pale-yellow liquid was obtained as a product. This product was used directly to the next step without further purification. The yellow liquid, **S5** was stirred overnight (15 h) together with 1.5 mL of oxalyl chloride in dichloromethane solvent. The volatiles were removed under reduced pressure and the residue was added drop by drop to 10 mL of ammonium hydroxide solution (28% m/m) with vigorous stirring while keeping the temperature below 10 °C. The reaction mixture was extracted with ethyl acetate and washed with brine. The organic layer was dried over Na_2SO_4 and concentrated under reduced pressure. The obtained crude solid was further purified by recrystallization from hexane/ethyl acetate solvent.

White solid (overall 59% yield, 2.1 g). m.p. 78-80 °C; ^{1}H-NMR (500 MHz, Methanol-d$_4$) δ 3.98 (s, 2H), 3.84 (t, *J* = 5.7 Hz, 2H), 3.56 (t, *J* = 5.7 Hz, 2H), 1.92 (s, 2H). ^{13}C{^{1}H}-NMR (125 MHz, CDCl$_3$) δ 172.0, 71.4, 70.2, 30.5. HRMS (ESI): cald for $C_4H_8BrNO_2$: m/z 181.9811 ([M+H]$^+$), found: m/z 181.9813.

2.3. General Procedure 2:

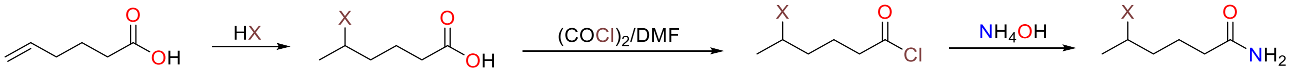

S7 **S8** **S9** **S10**

A mixture of alkenyl carboxylic acid, **S7** (1 g), 48% HBr (15 mL), or Conc. HCl (20 mL) was heated under reflux until the completion of the reaction. After cooling to room temperature, 100 mL of water was added to the reaction mixture. The reaction mixture was extracted several times with diethyl ether. The combined organic layer was dried over anhydrous Na_2SO_4 and evaporated in a vacuum. A pale-yellow liquid was obtained as a crude product (**S8**). This crude product was used directly to the next step without further purification. The yellow liquid, **S8** was stirred overnight (15 h) together with 1.5 mL of oxalyl chloride in dichloromethane solvent (20 mL). The excess of oxalyl chloride was removed under reduced pressure and the residue was added drop by drop to 20 mL of ammonium hydroxide solution (28% m/m) with vigorous stirring while keeping the temperature below 10 °C. The reaction mixture was extracted with ethyl acetate and washed with brine. The organic layer was dried over Na_2SO_4 and concentrated under reduced pressure. The obtained crude solid was further purified by recrystallization from hexane/ethyl acetate solvent.

5-bromohexanamide (4g)

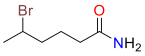

White solid (overall 22% yield, 456 mg). m.p. 54-56 °C; ^{1}H-NMR (500 MHz, Methanol-d$_4$) δ 4.16 (td, *J* = 13.0, 6.5 Hz, 1H), 2.17-2.21 (m, 2H), 1.77-1.82 (m, 3H), 1.67-1.74 (m, 4H). ^{13}C{^{1}H}-NMR (125 MHz, $CDCl_3$) δ 175.2, 51.1, 40.4, 35.0, 26.5, 23.7. HRMS (ESI): Cald for $C_6H_{12}BrNO$: m/z ([M]$^+$), 193.0102, found: 193.0118.

5-bromohexanamide (4g')

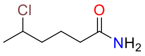

White solid (overall 15% yield, 195 mg). m.p. 81-82 °C; ^{1}H-NMR (500 MHz, Methanol-d$_4$) δ 3.94-4.00 (m, 1H), 2.10-2.14 (m, 2H), 1.58-1.75 (m, 4H), 1.40 (d, *J* = 6.9 Hz, 3H). ^{13}C{^{1}H}-NMR (125 MHz, $CDCl_3$) δ 174.8, 58.4, 39.6, 35.1, 25.4, 22.6. HRMS (ESI): cald for $C_6H_{12}ClNO$: m/z 172.0500 ([M+Na]$^+$), found: m/z 172.0500.

3. Optimization of Different Boron Source

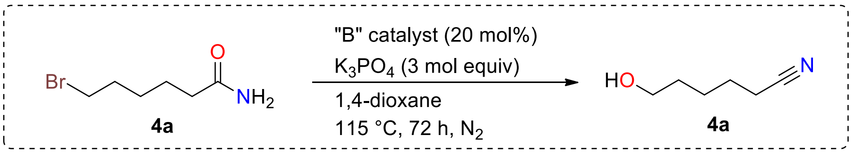

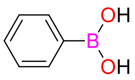

2a
80%

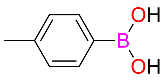

2b
78%

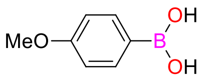

2c
71%

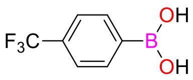

2d
54%

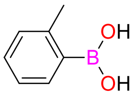

2e
61%

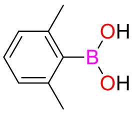

2f
42%

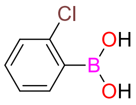

2g
59%

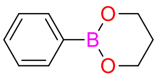

7
72%

4. Catalysis

4.1. General Procedure for Metal Free Dehydration of Amide

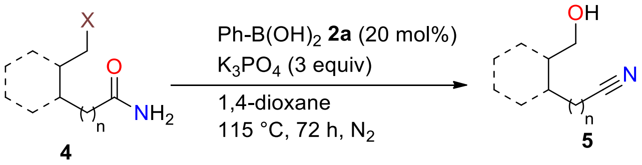

A mixture of phenylboronic acid **2a** (20 mol%, 24.2 mg), amide substrates **4** (1 mol equiv, 1 mmol), and K_3PO_4 (3 mol equiv, 636 mg) were added to a reaction tube. The reaction tube was degassed under vacuum and refilled with N_2 under standard Schlenk techniques (3 times). To the reaction mixture, 1,4-dioxane was added (2 mL). The reaction tube was sealed with a screw cap and teflon then placed over a chemiStation (metal block) under nitrogen for 72 h. Finally, the reaction was quenched with 1N HCl and extracted with EtOAc. The EtOAc layer was collected and dried over Na_2SO_4. The solvent was evaporated under vacuum and crude mass was purified by column chromatography (hexane/ethyl acetate) to give product **5**.

Table S-1. Deviation of the Reaction Conditions (Appendix)

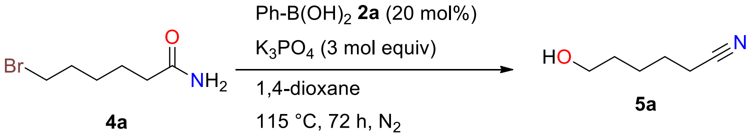

entry	deviation from standard conditions	yield (%)
1	none	80
2	10 mol% of **2a** instead of 20 mol%	60
3	without **2a**	0
4	toluene instead of 1,4-dioxane	72
5	KO*t*Bu instead of K_3PO_4	0
6	1 mol equiv of K_3PO_4 instead of 3 mol equiv	40
7	90 °C instead of 115 °C	20
8	24 h instead of 72 h	49

4.2. Characterization of Products

6-hydroxyhexanenitrile (5a, Ref. 9**)**

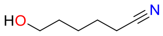

Colorless liquid (80% yield (91 mg) from **1a**, 47% yield (53 mg) from **1a'** and 74% yield (84 mg) from **1a''**). ^{1}H-NMR (500 MHz, Methanol-d_4) δ 3.56 (t, *J* = 6.3 Hz, 2H), 2.45 (t, *J* = 7.2 Hz, 2H), 1.64-1.69 (m, 2H), 1.47-1.59 (m, 4H). ^{13}C{^{1}H}-NMR (125 MHz, $CDCl_3$) δ 119.7, 62.4, 31.8, 25.3, 25.1, 17.3.

7-hydroxyheptanenitrile (5b, Ref. 10**)**

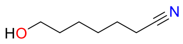
Colorless liquid (39% yield, 50 mg). ^{1}H-NMR (500 MHz, Methanol-d_4) δ 3.55 (t, *J* = 6.6 Hz, 2H), 2.43 (t, *J* = 7.2 Hz, 2H), 1.62-1.68 (m, 2H), 1.46-1.58 (m, 6H). ^{13}C{^{1}H}-NMR (125 MHz, Methanol-d_4) δ 119.8, 61.2, 31.4, 25.0, 24.8, 16.0.

4-hydroxybutanenitrile (5e, Ref. 11**)**

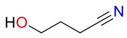
Colorless liquid (60% yield, 52 mg). ^{1}H- NMR (500 MHz, Methanol-d_4) δ 3.62 (t, *J* = 6.8 Hz, 2H), 2.50 (t, *J* = 6.8 Hz, 2H), 1.78-1.83 (m, 2H). ^{13}C{^{1}H}-NMR (125 MHz, $CDCl_3$) δ 119.6, 60.5, 28.1, 13.9.

5-hydroxypentanenitrile (5c, Ref. 12**)**

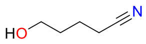
Colorless liquid (86% yield, 86 mg). ^{1}H-NMR (500 MHz, Methanol-d_4) δ 3.59 (t, *J* = 6.4 Hz, 2H), 2.47 (t, *J* = 6.4 Hz, 2H), 1.62-1.75 (m, 4H). ^{13}C{^{1}H}-NMR (125 MHz, $CDCl_3$) δ 119.6, 61.8, 31.4, 22.1, 17.1.

2-(2-hydroxyethoxy)acetonitrile (5d, Ref. 13**)**

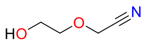
Colorless liquid (75%, 76 mg). ^{1}H-NMR (500 MHz, $CDCl_3$) δ 4.33 (s, 2H), 3.81 (t, *J* = 4 Hz, 2H), 3.72 (t, *J* = 4.0 Hz, 2H), 1.86 (brs, 1H). ^{13}C{^{1}H}-NMR (125 MHz, $CDCl_3$) δ 115.7, 72.7, 61.5, 56.4.

((*cis*)-2-cyanocyclohexyl)methyl 4-methylbenzenesulfonate (S11)

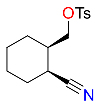
The obtained crude product ((*cis*)-2-(hydroxymethyl)cyclohexane-1-carbonitrile, **5h**) was passed through short column chromatography to remove the phenylboronic acid derivatives. The obtained product contained unknown impurities. Then the liquid product was further reacted with tosyl chloride and pyridine at room temperature for overnight (15 h) in dichloromethane solvent (5 mL). The reaction mixture was extracted with dichloromethane and Water. The organic layer was dried over Na_2SO_4 and the volatiles were evaporated by using a rotary vacuum. The crude mass was purified by column chromatography with hexane and ethyl acetate (10:1) as eluent. The compound was obtained as a single diastereomer. Colorless liquid (63%, 184 mg). ^{1}H-NMR (500 MHz, $CDCl_3$) δ 7.79 (d, *J* = 8.0 Hz, 2H), 7.36 (d, *J* = 8.6 Hz, 2H), 4.05-4.12 (m, 2H), 2.45 (s, 3H), 2.38 (td, *J* = 11.3, 3.6 Hz, 1H), 2.09-2.12 (m, 1H), 1.75-1.83 (m, 4H), 1.51-1.59 (m, 1H), 1.16-1.32 (m, 3H). ^{13}C{^{1}H}-NMR (125 MHz, $CDCl_3$) δ 145.2, 132.4, 130.0, 128.0, 120.7, 71.7, 39.6, 30.0, 29.6, 27.8, 24.4, 21.7. Elemental analysis for $C_{15}H_{19}NO_3S$; C (cald: 61.41; obtained: 61.40), H (cald: 6.53; obtained: 6.53), N (cald: 4.77; obtained: 4.75); S (cald: 10.93; obtained: 10.95).

5-hydroxyhexanenitrile (5g, Ref. 14**)**

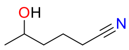
The reaction was performed in 0.57 mmol scale with **4g** and 1 mmol scale for **4g'**. Colorless liquid (91% yield (59 mg) from **4g** and 82% yield (93 mg) from **4g'**). ^{1}H-NMR (500 MHz, Methanol-d_4) δ 3.72-3.78 (m, 1H), 2.42-2.49 (m, 2H), 1.64-1.80 (m, 2H), 1.51-1.56 (m, 2H), 1.17 (d, *J* = 6.3 Hz, 3H). ^{13}C{^{1}H}-NMR (125 MHz, $CDCl_3$) δ 119.8, 67.3, 37.8, 23.9, 21.9, 17.3.

5. DFT Studies for Probable Reaction Mechanism

Computational details

All calculations were carried out at the density functional theory (DFT) level using M06-2X functional as implemented in Gaussian 16.[15] The 6-311+G(d,p) basis set was used for all atoms. The solvent effect was taken into account by the self-consistent reaction field (SCRF) method. The geometries of local minima and transition states were optimized without symmetry restrictions and confirmed by the number of imaginary frequencies. The intrinsic reaction coordinate (IRC) method was used to track minimum energy paths from transition state structures to the corresponding local minima. Substrate **4a** was assumed to exist in the anionic deprotonated form under the experimental conditions. Initially, we mapped the electrostatic potentials (ESP) of the deprotonated form of **4a**, which suggests that oxygen atom is more electron rich compared to the nitrogen atom (Figure S1). So, the coordination between phenylboronic acid (**2a**) and **4a** might take place via oxygen rather than the nitrogen.

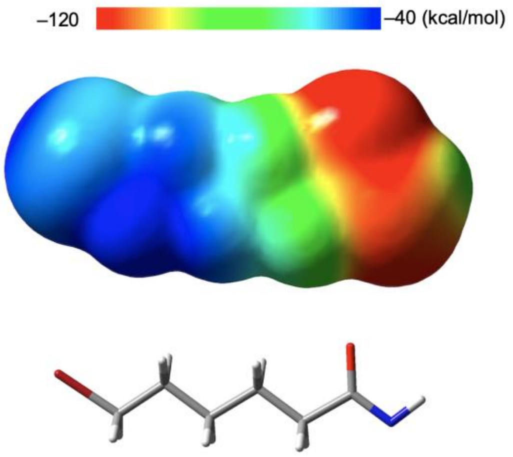

Figure S1. Electrostatic Potential (ESP) Map of the Deprotonated Form of Substrate **4a** (isovalue = 0.0004).

As shown in Figure S2, our calculations suggested that the reaction proceeds through a two-step mechanism. First, a deprotonated amide substrate interacts with boronic acid **2a** to form a tetrahedral boron intermediate **A**. An intramolecular nucleophilic substitution of the bromine with one OH group then occurs via the transition state **TS1**, which results in the intermediate **B**. The transition state was calculated to be 21.0 kcal/mol higher in energy compared to intermediate **A**. The second step is the cleavage of the C-O bond. We found that K_2HPO_4 plays an essential role in the second step. One oxygen atom of K_2HPO_4 is coordinated to the boron atom in intermediate **B'** where NH and POH protons are directed towards the PO and CO oxygen atoms, respectively. The NH and POH protons simultaneously transfer through a single

transition state (**TS2**) to give the corresponding hydroxyalkyl nitrile (intermediate **C**). The activation energy for **TS2** (26.7 kcal/mol) was slightly higher than that of **TS1**.

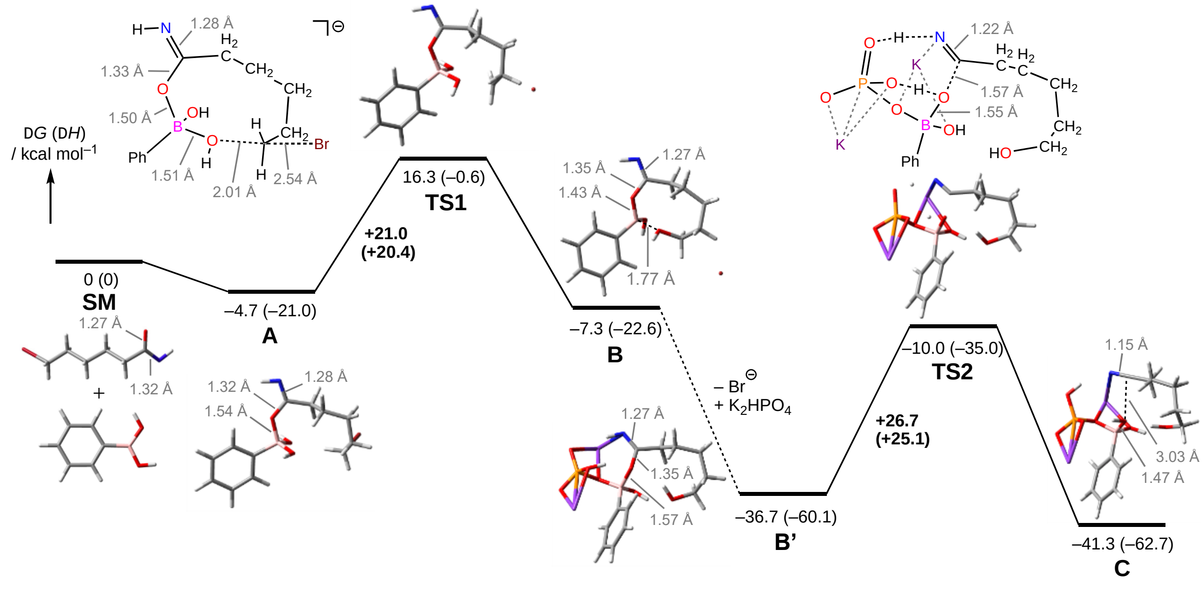

Figure S2. Calculated Reaction Energy Diagram for the Oxygen Transfer Reaction.

6. List of Side Reaction Products

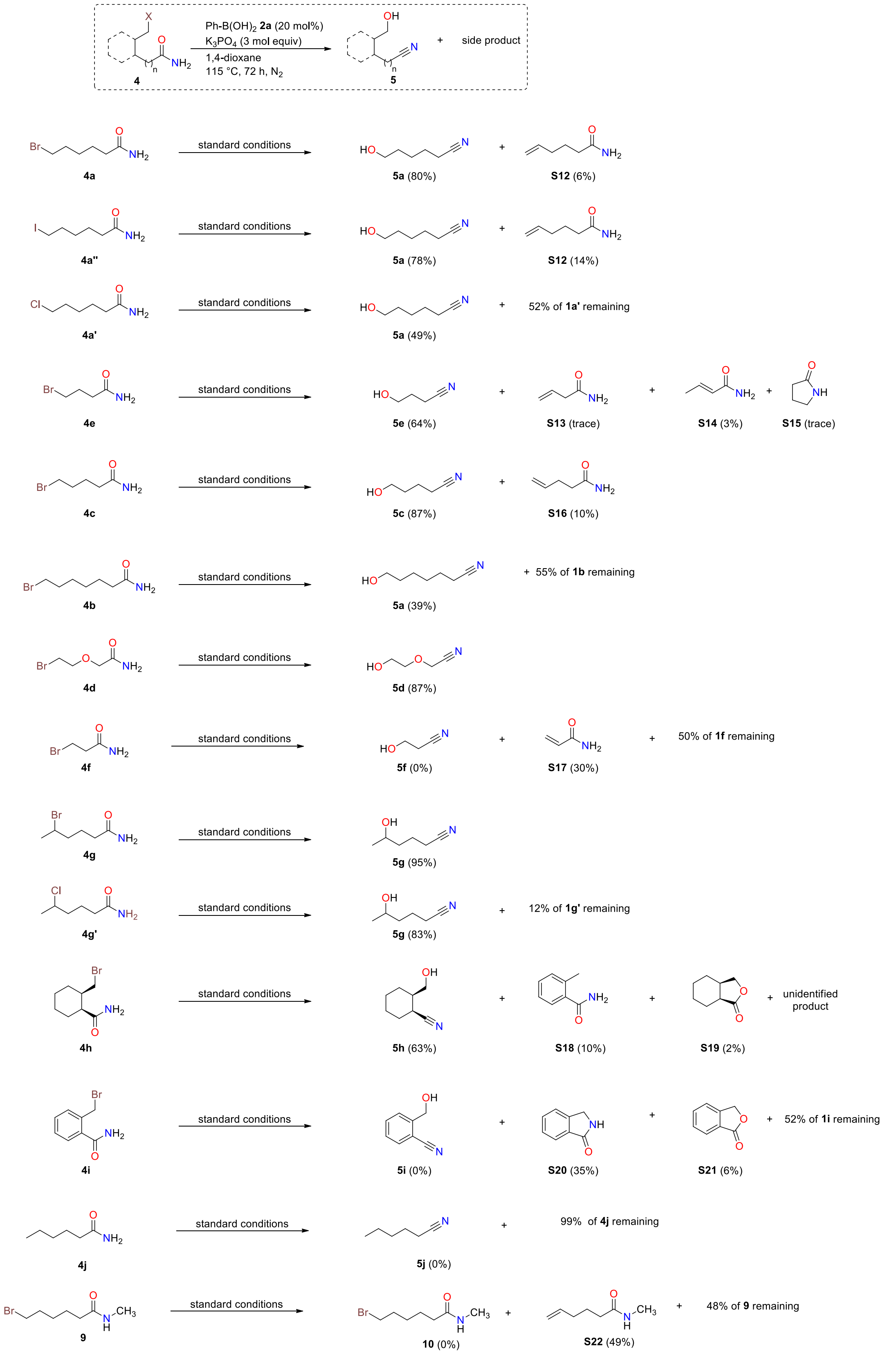

7. Supplementary References

1. Deane, F. M.; O'Sullivan, E. C.; Maguire, A. R.; Gilbert, J.; Sakoff, J. A.; McCluskey, A.; McCarthy, F. O. Synthesis and evaluation of novel ellipticines as potential anti-cancer agents. *Org. Biomol. Chem.* **11**, 1334–1344 (2013).
2. Yoshimura, A.; Middleton, K. R.; Luedtke, M. W.; Zhu, C.; Zhdankin, V. V. Hypervalent iodine catalyzed hofmann rearrangement of carboxamides using oxone as terminal oxidant. *J. Org. Chem.* **77**, 11399–11404 (2012).
3. Kohda, I.; Iwai, M.; Watanabe, M.; Arakawa, Y.; Fukaya, C.; Yokoyama, K.; Kohama, Y.; Mimura, T. Inhibitory effect of 2-(*E*-2-alkenoylamino)ethyl alkyl sulfides on gastric ulceration in rats. II. Structure and activity relationships of 2-(*E*-n or *Z*-n-decenoylamino)ethyl alkyl sulfides. *Chem. Pharm. Bull.* **39**, 1546–1550 (1991).
4. Fries, R. W.; Bohlken, D. P.; Blakley, R. T.; Plapp, B. V. Activation of liver alcohol dehydrogenases by imidoesters generated in solution. *Biochemistry* **14**, 5233–5238 (1975).
5. Li, X.; Singh, S. M.; Labrie, F. A convenient and practical method for conversion of primary alkyl chlorides to highly pure bromides (≥99%). *Synth. Commun.* **24**, 733–743 (1994).
6. Henri, C.; Alain, D.; Francoise, P. Diels-Alder reactions of γ-substituted crotononitriles and esters. II. Reactions of the addition compounds. *Bull. Soc. Chim. Fr.* **13**, 2535–2541 (1966).
7. Shaw, S. J.; Chen, Y.; Zheng, H.; Fu, H.; Burlingame, M. A.; Marquez, S.; Li, Y.; Claypool, M.; Carreras, C. W.; Crumb, W.; Hardy, D. J.; Myles, D. C.; Liu, Y. Structure−activity relationships of 9-substituted-9-dihydroerythromycin-based motilin agonists: optimizing for potency and safety. *J. Med. Chem.* **52**, 6851–6859 (2009).
8. Wang, Q., Tang, X.-T., Shi, M. Metal-free cross-coupling of arylboronic acids and derivatives with DAST-type reagents for direct access to diverse aromatic sulfonamides and sulfonamide. *Angew. Chem. Int. Ed.* **55**, 10811–10815 (2016).
9. Wang, G.; Xie, X.; Xu, W.; Liu, Y. Nickel-catalyzed highly regioselective hydrocyanation of alkenes with $Zn(CN)_2$. *Org. Chem. Front.* **6**, 2037–2042 (2019).
10. Wulkesch, C.; Czekelius, C. Straightforward synthesis of fluorinated enals via photocatalytic α-perfluoroalkenylation of aldehydes. *J. Org. Chem.* **86**, 7425–7438 (2021).
11. Fukuyama, T.; Yamada, K.; Nishikawa, T.; Ravelli, D.; Fagnoni, M.; Ryu, I. Site-selectivity in TBADT-photocatalyzed C(sp^3)-H functionalization of saturated alcohols and alkanes. *Chem. Lett.* **47**, 207–209 (2018).
12. Bao, X.; Wang, Q.; Zhu. J. Dual photoredox/copper catalysis for the remote C(sp^3)−H functionalization of alcohols and alkyl halides by *N*-alkoxypyridinium salts. *Angew. Chem., Int. Ed.* **58**, 2139–2143 (2019).

13. Zimmerman, S. C.; Cramer, K. D.; Galan, A. A. Synthesis of 2,4(5)-bis(hydroxymethyl)imidazoles and 2, 4(5)-bis[(2-hydroxyethoxy)methyl]imidazoles. Precursors of 2,4(5)-connected imidazole crown ethers. *J. Org. Chem.* **54**, 1256–264 (1989).

14. Edina L.; Vitomir Š. Chemoenzymatic synthesis of (2R)-2-hydroxyundecan-6-one. *Synthesis*. **2001**, 423–426 (2001).

15. Gaussian 16, Revision C.01, M. J. Frisch, G. W. Trucks, H. B. Schlegel, G. E. Scuseria, M. A. Robb, J. R. Cheeseman, G. Scalmani, V. Barone, G. A. Petersson, H. Nakatsuji, X. Li, M. Caricato, A. V. Marenich, J. Bloino, B. G. Janesko, R. Gomperts, B. Mennucci, H. P. Hratchian, J. V. Ortiz, A. F. Izmaylov, J. L. Sonnenberg, D. Williams-Young, F. Ding, F. Lipparini, F. Egidi, J. Goings, B. Peng, A. Petrone, T. Henderson, D. Ranasinghe, V. G. Zakrzewski, J. Gao, N. Rega, G. Zheng, W. Liang, M. Hada, M. Ehara, K. Toyota, R. Fukuda, J. Hasegawa, M. Ishida, T. Nakajima, Y. Honda, O. Kitao, H. Nakai, T. Vreven, K. Throssell, J. A. Montgomery, Jr., J. E. Peralta, F. Ogliaro, M. J. Bearpark, J. J. Heyd, E. N. Brothers, K. N. Kudin, V. N. Staroverov, T. A. Keith, R. Kobayashi, J. Normand, K. Raghavachari, A. P. Rendell, J. C. Burant, S. S. Iyengar, J. Tomasi, M. Cossi, J. M. Millam, M. Klene, C. Adamo, R. Cammi, J. W. Ochterski, R. L. Martin, K. Morokuma, O. Farkas, J. B. Foresman, D. J. Fox, Gaussian, Inc., Wallingford CT, 2019.
