# Supplementary material for: Oxygen transfer reaction of haloalkyl amides catalyzed by phenylboronic acid
Source: Commun Chem. 2023 Feb 10;6:29. doi: 10.1038/s42004-023-00824-6 (PMC9918490; doi:10.1038/s42004-023-00824-6)
Supplement: Supplementary file 2 — Description of Additional Supplementary Files [file 42004_2023_824_MOESM2_ESM.pdf]

Description of Additional Supplementary Files

File name: Supplementary Data 1

Description: The cartesian coordinates for the DFT studies.

File name: Supplementary Data 2

Description: The NMR and GC spectra data.
