# Supplementary material for: Oxygen transfer reaction of haloalkyl amides catalyzed by phenylboronic acid
Source: Commun Chem. 2023 Feb 10;6:29. doi: 10.1038/s42004-023-00824-6 (PMC9918490; doi:10.1038/s42004-023-00824-6)
Supplement: Supplementary file 3 — Supplementary Data 1 [file 42004_2023_824_MOESM3_ESM.pdf]

Supporting Information (Supplementary Data 1)

Oxygen Transfer Reaction of Haloalkyl Amides Catalyzed by Phenylboronic Acid

Abhijit Sen, Atsuya Muranaka, Aya Ohno, and Yoichi M. A. Yamada*

RIKEN Center for Sustainable Resource Science, Wako, Saitama 351-0198, Japan

ymayamada@riken.jp

Cartesian Coordinates (in Å) and Energies

4a

E(RM062X) = -2939.450369 A.U.

C	1.94359900	-0.72294000	-0.32211800
H	1.98345700	-1.65771200	0.23293900
H	2.01341200	-0.92584300	-1.38856200
C	0.72227700	0.10062700	0.02803700
H	0.75735800	1.05627800	-0.50234800
H	0.72525700	0.32410200	1.09863600
C	-0.55813500	-0.65357500	-0.34035300
H	-0.56776600	-1.62391400	0.17078900
H	-0.56000700	-0.86757900	-1.41616900
C	-1.82495600	0.11988400	0.02158800
H	-1.83994500	1.08487200	-0.49386600
H	-1.82093600	0.33759900	1.09634400
C	-3.09959100	-0.64489200	-0.32836600
H	-3.11396000	-1.61278400	0.17812200
H	-3.11932700	-0.82472800	-1.41050000
Br	3.61899800	0.21124400	0.13077800
C	-4.36211500	0.14585500	0.04215800
O	-4.46027200	1.30522400	-0.45565500
N	-5.19814000	-0.47954400	0.84264600
H	-5.97979800	0.16143100	0.99843700

2a

E(RM062X) = -408.228332 A.U.

C	-0.17484300	0.01803300	-0.00001000
C	0.52816700	-1.19354500	0.00007900
C	0.56463100	1.20619900	-0.00010600
C	1.91826600	-1.21788800	0.00009100
H	-0.02990300	-2.12316000	0.00013900
C	1.95551400	1.19181200	-0.00010100
H	0.06281100	2.17002900	-0.00020600
C	2.63371400	-0.02344200	0.00000000
H	2.44494600	-2.16502400	0.00016600
H	2.50902900	2.12335100	-0.00018300
H	3.71747100	-0.03904300	0.00000500
B	-1.74219600	-0.00001900	-0.00002500
O	-2.52363400	1.12359300	0.00017600
O	-2.37348200	-1.20900600	-0.00016200
H	-3.33153400	-1.12036000	-0.00009100
H	-2.03760800	1.95058600	0.00046000

A

E(RM062X) = -3347.714615 A.U.

N	-0.35207200	3.52337300	-1.07899000
H	-1.34825800	3.73490300	-1.16131200
O	-1.29096100	1.52766200	-0.48732300
C	-0.23721900	2.30259700	-0.69779500
C	2.31414500	-1.15004700	0.82347000
H	2.32627300	-2.14539000	1.26147200
H	1.32891800	-0.94802600	0.41390600
C	2.80143500	-0.11261400	1.81806000
H	3.76006700	-0.44615700	2.22806500
H	2.07290800	-0.13909300	2.63855600
C	2.94098300	1.33630100	1.33712300
H	3.63125300	1.37742300	0.48695100
H	3.42221200	1.89142100	2.14886500
C	1.62473900	2.03492300	0.97588100
H	1.74583800	3.11748600	1.07614500
H	0.84909100	1.72393500	1.67919300
C	1.15178900	1.75188100	-0.45747800
H	1.15503100	0.68812400	-0.67625500
H	1.82961000	2.24907000	-1.15480000
Br	3.52171400	-1.30154900	-0.73737800
C	-2.88673100	-0.34612400	-0.00671300
C	-3.93947200	0.57312500	-0.08223100
C	-3.23559100	-1.69578000	0.15300000
C	-5.27384000	0.17239000	-0.00024600
H	-3.69998000	1.62283700	-0.21650900
C	-4.56186800	-2.11153700	0.23406600
H	-2.43720500	-2.43077700	0.20845400
C	-5.59071500	-1.17345500	0.15889900
H	-6.06789400	0.90978000	-0.06469500
H	-4.79780700	-3.16412600	0.35515600
H	-6.62653600	-1.48966500	0.22098300
B	-1.30156100	0.05638500	-0.04804000
O	-0.66609000	-0.10495200	1.26022100
O	-0.53144400	-0.78628400	-0.95848800
H	-0.92508200	-0.77301900	-1.83220500
H	-1.24408800	0.24858100	1.93789500

TS1

E(RM062X) = -3347.680982 A.U.

N	-1.21031600	3.88823000	-0.91431600
H	-2.23157500	3.86251000	-0.90272500
O	-1.60458100	1.69693300	-0.39926600
C	-0.77921800	2.71300800	-0.65117000
C	1.63788800	-0.61398900	0.48577800
H	1.36955700	-1.63308400	0.71325300
H	1.52631600	-0.30947300	-0.53881100
C	2.18082100	0.27090200	1.57523000
H	3.09536500	-0.19010200	1.94686800
H	1.46361100	0.24841700	2.40207100
C	2.45746800	1.72380800	1.18139200
H	3.12314100	1.73808500	0.31065900
H	3.01600900	2.17777200	2.00508200
C	1.19664600	2.55731500	0.90785300
H	1.38719600	3.60869000	1.13900900

H	0.40151000	2.22532200	1.57968400
C	0.70740200	2.47310800	-0.55198000
H	0.93459300	1.50390400	-0.98861200
H	1.21332400	3.23265000	-1.14900300
Br	3.83585500	-1.57572600	-0.34963700
C	-2.62820400	-0.57156300	-0.10250600
C	-3.77768100	0.03180700	0.42132000
C	-2.68432000	-1.95100200	-0.34698300
C	-4.93030900	-0.70385400	0.69280100
H	-3.76266100	1.10053400	0.60808700
C	-3.82947100	-2.69787900	-0.07985900
H	-1.81025700	-2.44271300	-0.76597500
C	-4.95950900	-2.07346200	0.44320400
H	-5.80804600	-0.20973800	1.09647800
H	-3.84460800	-3.76361700	-0.28292500
H	-5.85510900	-2.64873600	0.65113200
B	-1.24789000	0.23761100	-0.39106400
O	-0.27564500	-0.03796300	0.73225900
O	-0.55762900	-0.17345500	-1.59994900
H	-1.11179900	-0.03396000	-2.36979500
H	-0.69608000	-0.55502200	1.42354100

B

E(RM062X) = -3347.719562 A.U.

N	-2.34691800	3.73914100	-0.80785100
H	-3.31927000	3.42873400	-0.77180200
O	-2.10222600	1.52244900	-0.30081600
C	-1.59079900	2.74329300	-0.58437000
C	1.23907800	0.08719700	0.53290900
H	1.52570000	-0.94354900	0.73807900
H	1.44296700	0.27973100	-0.51843900
C	1.95615400	1.05298500	1.45773600
H	3.00556700	0.74523900	1.45941000
H	1.57747800	0.89895700	2.47642300
C	1.85414300	2.53895000	1.09321600
H	2.41868900	2.72039000	0.17163700
H	2.36249400	3.10552700	1.87859600
C	0.42151300	3.07025500	0.92009600
H	0.38074000	4.13182600	1.17518700
H	-0.24598200	2.55824700	1.61834600
C	-0.09523000	2.90576700	-0.52648100
H	0.37484700	2.05150700	-1.00957600
H	0.16393300	3.79089000	-1.10749500
Br	4.22930400	-1.46363200	-0.34541900
C	-2.36817500	-0.98029500	-0.14150300
C	-3.72887200	-0.83684000	0.14733500
C	-1.82284400	-2.27023600	-0.08072200
C	-4.51875200	-1.93638200	0.47582300
H	-4.17011200	0.15331300	0.10985100
C	-2.60245100	-3.37511400	0.24896200
H	-0.76457400	-2.41857000	-0.28729900
C	-3.95675800	-3.20860100	0.52776100
H	-5.57266400	-1.80143000	0.69287800
H	-2.15528100	-4.36186200	0.29116100
H	-4.56879700	-4.06562900	0.78513800
B	-1.46070500	0.26902400	-0.56143600

O	-0.20682300	0.21353000	0.68796400
O	-0.73745400	0.21304800	-1.76174700
H	-0.59103800	-0.68290400	-2.07161200
H	-0.50843500	-0.22051700	1.49355600

B’

E(RM062X) = -2616.433453 A.U.

N	1.12947200	-2.61504300	-1.01125400
H	0.13505900	-2.57463500	-1.25228600
O	0.82846700	-0.33699600	-0.62830700
C	1.56889600	-1.46356700	-0.68482400
C	4.05632800	1.60549400	1.05135300
H	4.32386600	2.46606000	1.66624400
H	4.27398000	0.70424800	1.64075600
C	4.89241500	1.60925600	-0.22607900
H	5.90100100	1.94101200	0.03983000
H	4.48050100	2.37165900	-0.89695700
C	5.01332400	0.27212900	-0.96823100
H	5.53991700	-0.44921300	-0.33219400
H	5.65458600	0.43514800	-1.83865100
C	3.68533100	-0.34266900	-1.43217500
H	3.84742900	-0.94626500	-2.32866800
H	2.99517200	0.45469300	-1.73138800
C	3.02440300	-1.24730400	-0.37452700
H	3.08031300	-0.79957700	0.62098000
H	3.53556500	-2.20995400	-0.34101000
C	-0.63359600	1.38792200	0.53564800
C	-0.32876900	2.28026800	-0.49901000
C	-1.56632200	1.81183100	1.49419100
C	-0.92322600	3.54091500	-0.57885900
H	0.38776400	1.98230500	-1.25847300
C	-2.16513000	3.06887000	1.42983100
H	-1.83401000	1.13309200	2.29818500
C	-1.84559500	3.93997400	0.38730100
H	-0.65991100	4.21557500	-1.38627500
H	-2.87662300	3.37214800	2.19018600
H	-2.30016600	4.92297500	0.33683000
B	-0.00273700	-0.09946800	0.68533000
O	2.65419700	1.73355800	0.83808500
O	0.81897200	-0.31631500	1.84212400
H	1.40575200	0.43806400	1.96792100
H	2.35706600	1.15955700	0.12059900
P	-2.21751600	-1.46704800	-0.30629100
O	-2.11994800	-2.94904900	-0.54441500
O	-3.51811500	-0.82678400	0.08114900
O	-1.04141300	-1.14518600	0.74434700
K	-3.38329000	1.46089800	-1.04572900
K	-0.29104500	-3.58936300	1.25956200
O	-1.74181700	-0.61463300	-1.64145400
H	-0.77587800	-0.50315900	-1.67190800

TS2

E(RM062X) = -2616.385431 A.U.

N	1.13995600	-2.66364100	-0.76388100
H	-0.28504800	-2.95538100	-0.91170100
O	0.74328500	-0.25663700	-0.54262600

C	1.62427300	-1.55448400	-0.63096800
C	3.88665400	1.83889600	0.93125300
H	4.10303200	2.71754100	1.54106600
H	4.33142200	0.97131300	1.43758500
C	4.51103600	2.00591200	-0.45171200
H	5.48471900	2.48700900	-0.31598500
H	3.89364300	2.71027900	-1.02142100
C	4.72541900	0.72264200	-1.26582400
H	5.42548800	0.06903000	-0.73161400
H	5.22389400	1.00519600	-2.19713100
C	3.45219500	-0.06680500	-1.60107900
H	3.60145300	-0.63550500	-2.52228500
H	2.63255500	0.62845500	-1.80992100
C	3.03973400	-1.05882500	-0.50049200
H	3.13756800	-0.60847500	0.49176900
H	3.69625800	-1.93026900	-0.52656900
C	-0.82306700	1.32958800	0.71136800
C	-0.52379900	2.34000800	-0.21054300
C	-1.90289700	1.55571000	1.57837700
C	-1.26899900	3.51816200	-0.27587800
H	0.29632000	2.19793300	-0.90733900
C	-2.65276300	2.72984900	1.52959700
H	-2.17210800	0.78021900	2.28913800
C	-2.34006000	3.71625100	0.59382900
H	-1.01247100	4.28282500	-1.00102300
H	-3.47965600	2.87681600	2.21593400
H	-2.91755500	4.63280900	0.55092100
B	-0.09238900	-0.12235100	0.76080900
O	2.46607200	1.73060600	0.92637600
O	0.73317400	-0.41224500	1.90782200
H	1.34367900	0.31938300	2.05916200
H	2.16215300	1.21878100	0.16540200
P	-1.90429100	-1.59722500	-0.62414900
O	-1.37328400	-3.05700700	-0.87954300
O	-3.38597000	-1.46301000	-0.47794000
O	-1.12863900	-1.19113700	0.73488900
K	-3.32544700	1.09554900	-1.25289700
K	0.24348100	-3.06781700	1.83966500
O	-1.31778000	-0.62157000	-1.69712600
H	-0.12257500	-0.37267900	-1.27160500

C

E(RM062X) = -2616.435894 A.U.

N	2.10445900	-2.79935000	-0.26166300
H	0.18354000	-3.08612900	-1.29304600
O	0.85087400	0.45454300	-0.37410600
C	2.77724700	-1.87380700	-0.13408800
C	3.33114800	2.95722300	0.43807800
H	2.81710100	3.92700800	0.41430300
H	4.20164700	3.06598200	1.09283800
C	3.79004300	2.61303600	-0.97446400
H	4.36802300	3.47147400	-1.33245300
H	2.91277800	2.54015200	-1.62880200
C	4.64679800	1.34471300	-1.13296600
H	5.34934700	1.26866600	-0.29404500
H	5.26041200	1.47506600	-2.02770900

C	3.87378900	0.02370400	-1.29572600
H	4.42359400	-0.64169900	-1.96568000
H	2.90185500	0.21465700	-1.75377300
C	3.64827300	-0.71579200	0.03217600
H	3.17388400	-0.05950400	0.76435600
H	4.59929900	-1.06861600	0.44135100
C	-1.33245400	1.10229600	0.80929700
C	-1.31240400	2.36589900	0.20424300
C	-2.51219400	0.74073100	1.47834600
C	-2.41281800	3.22501600	0.24835600
H	-0.41779900	2.67543800	-0.32825700
C	-3.61822800	1.58724500	1.53686700
H	-2.57312500	-0.24364500	1.93208600
C	-3.57423000	2.83541300	0.91343800
H	-2.36490800	4.19701200	-0.23132300
H	-4.51612700	1.27531500	2.05969500
H	-4.43060200	3.49919600	0.95544300
B	-0.08250800	0.04521800	0.68369200
O	2.49700200	1.97833500	1.02368800
O	0.65734000	-0.16044300	1.94052400
H	1.13949800	0.63704000	2.18131800
H	1.89051600	1.60597400	0.34697800
P	-1.42952300	-1.67385500	-0.95931100
O	-0.78364800	-3.12060700	-1.31946300
O	-2.90178500	-1.86064900	-0.71974400
O	-0.64863500	-1.32345600	0.39563100
K	-3.39073400	0.61104100	-1.55697900
K	0.50807400	-2.74786200	2.10351100
O	-1.07691400	-0.62285000	-2.00087600
H	0.39207800	0.33033100	-1.22110700
