# Supplementary material for: Oxygen transfer reaction of haloalkyl amides catalyzed by phenylboronic acid
Source: Commun Chem. 2023 Feb 10;6:29. doi: 10.1038/s42004-023-00824-6 (PMC9918490; doi:10.1038/s42004-023-00824-6)
Supplement: Supplementary file 4 — Supplementary Data 2 [file 42004_2023_824_MOESM4_ESM.pdf]

Supporting Information (Supplementary data 2)

Oxygen Transfer Reaction of Haloalkyl Amides Catalyzed by Phenylboronic Acid

Abhijit Sen, Atsuya Muranaka, Aya Ohno, and Yoichi M. A. Yamada*

RIKEN Center for Sustainable Resource Science, Wako, Saitama 351-0198, Japan

ymayamada@riken.jp

1. NMR Spectra of Starting Materials

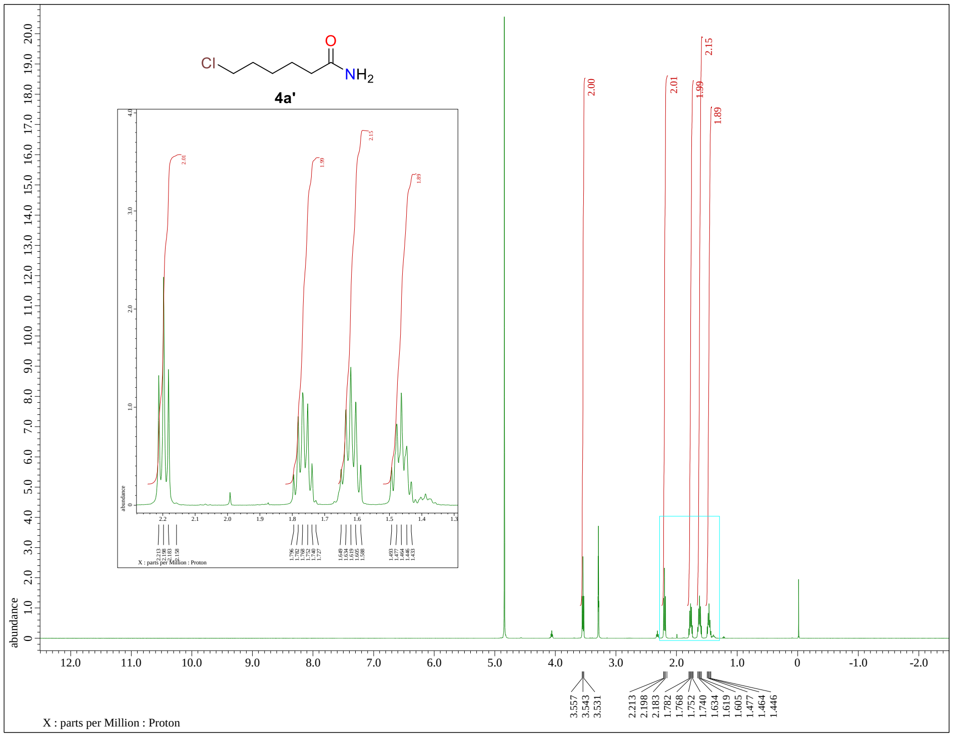

Figure S3-1. ^{1}H NMR spectrum of compound **4a'** (500 MHz, CD_3OD)

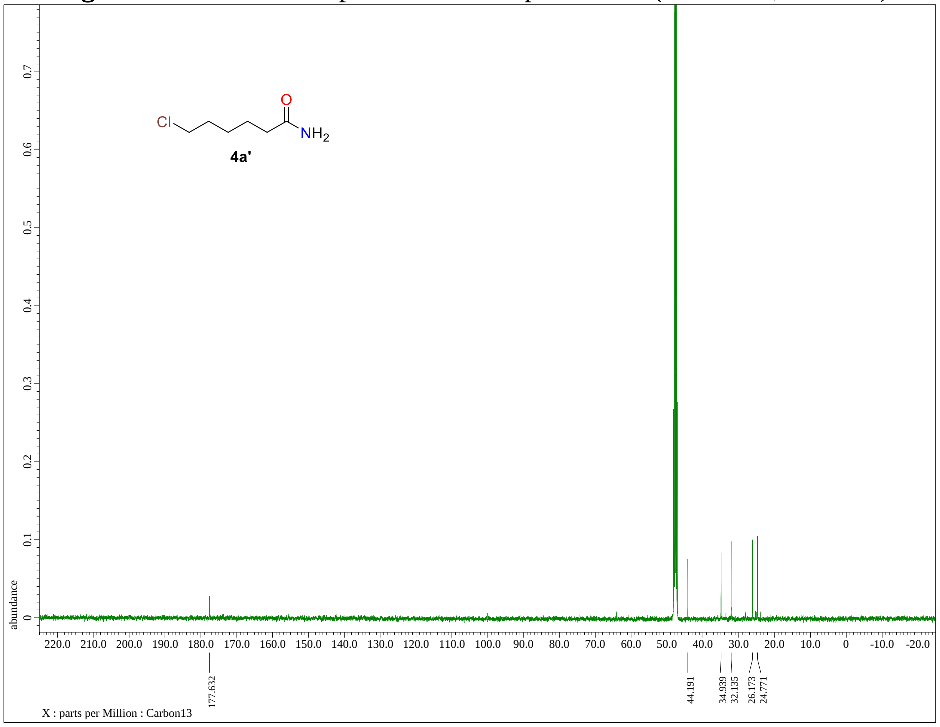

Figure S3-2. ^{13}C NMR spectrum of compound **4a'** (125 MHz, CD_3OD)

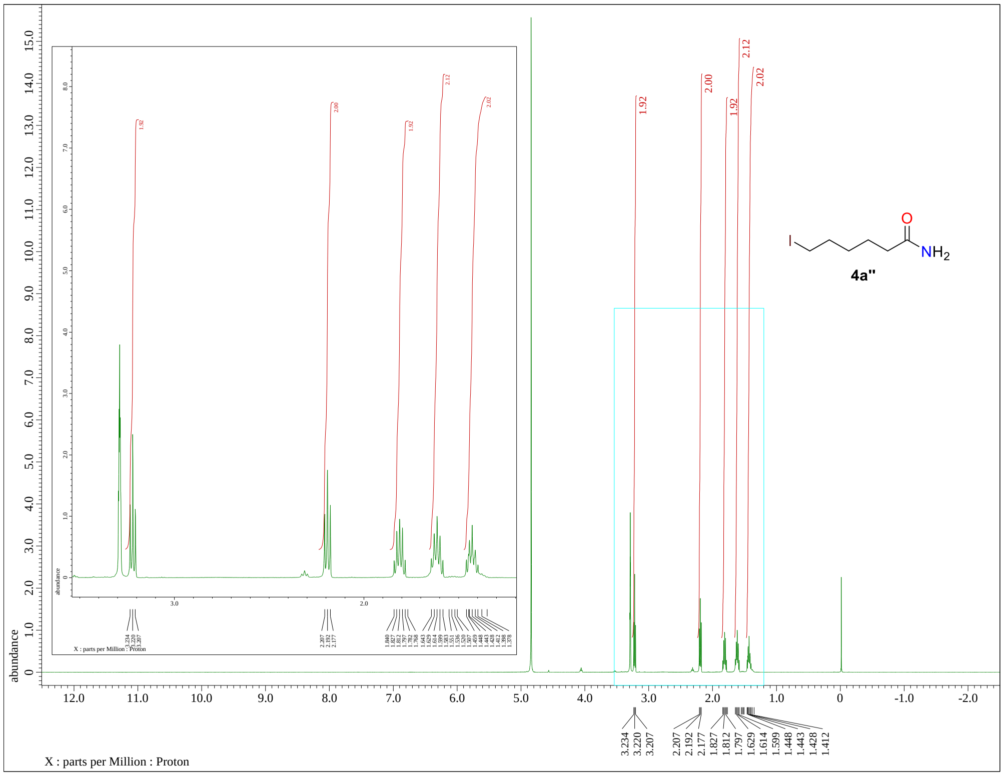

Figure S4-1. ^{1}H NMR spectrum of compound **4a''** (500 MHz, CD_3OD)

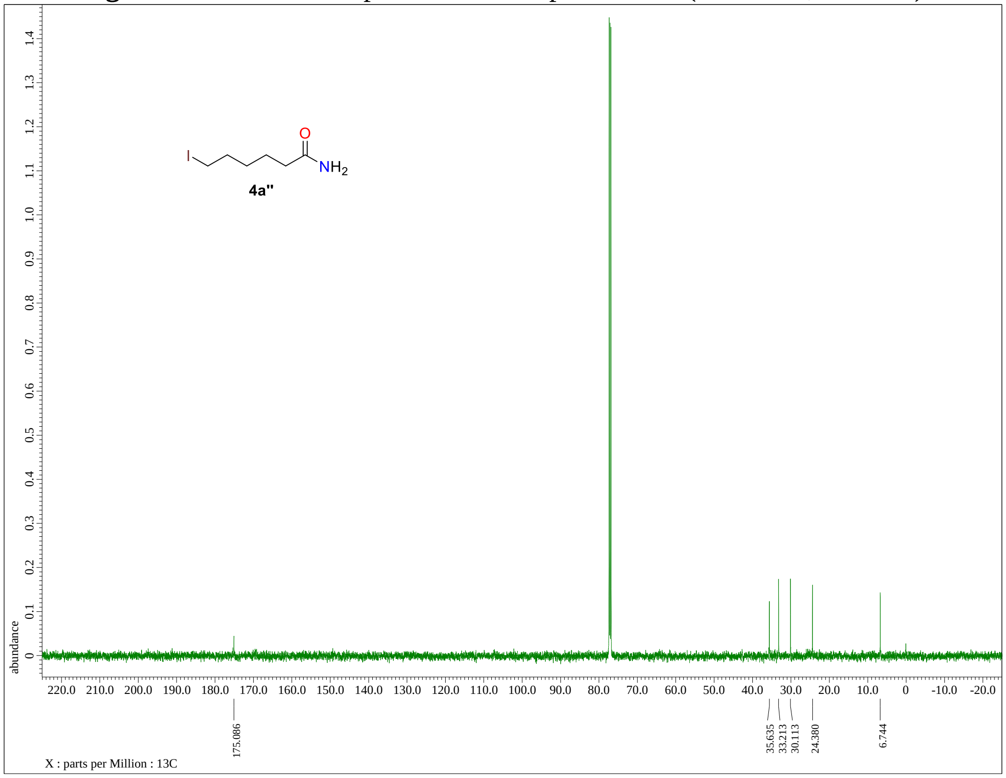

Figure S4-2. ^{13}C NMR spectrum of compound **4a''** (125 MHz, $CDCl_3$)

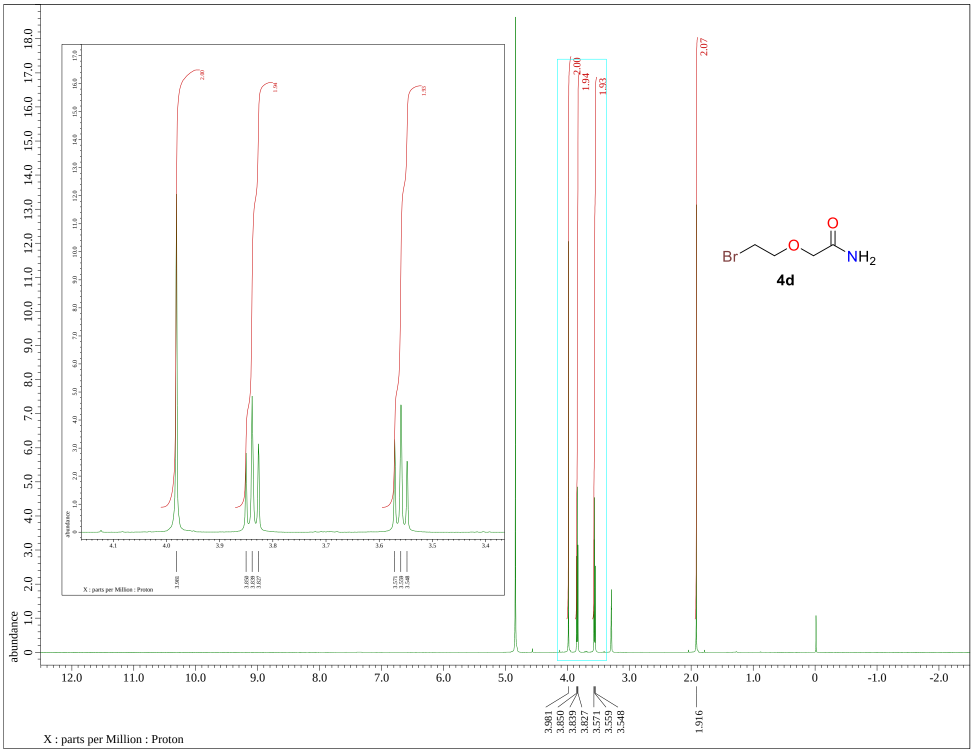

Figure S5-1. ^{1}H NMR spectrum of compound **4d** (500 MHz, CD_3OD)

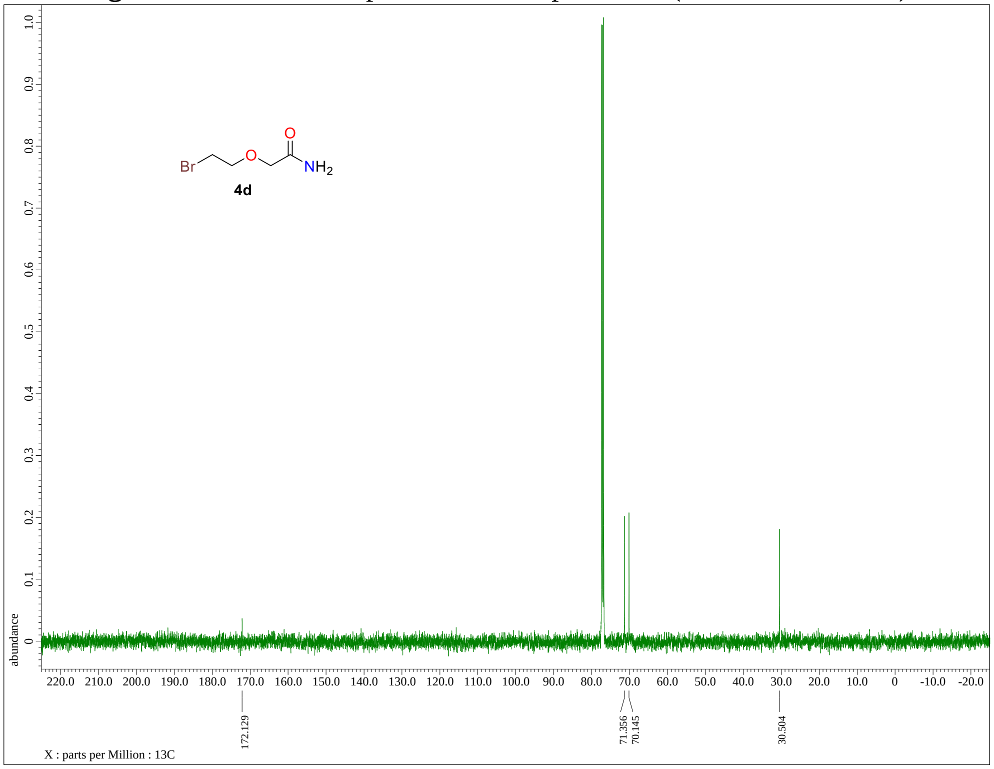

Figure S5-2. ^{13}C NMR spectrum of compound **4d** (125 MHz, $CDCl_3$)

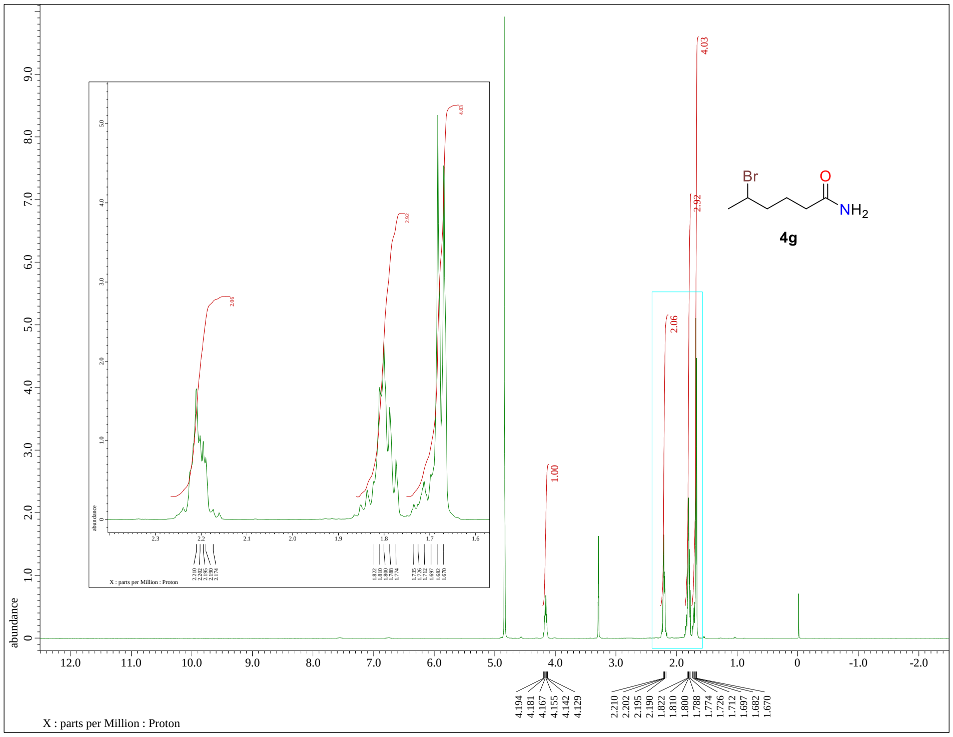

Figure S6-1. ^{1}H NMR spectrum of compound **4g** (500 MHz, CD3OD)

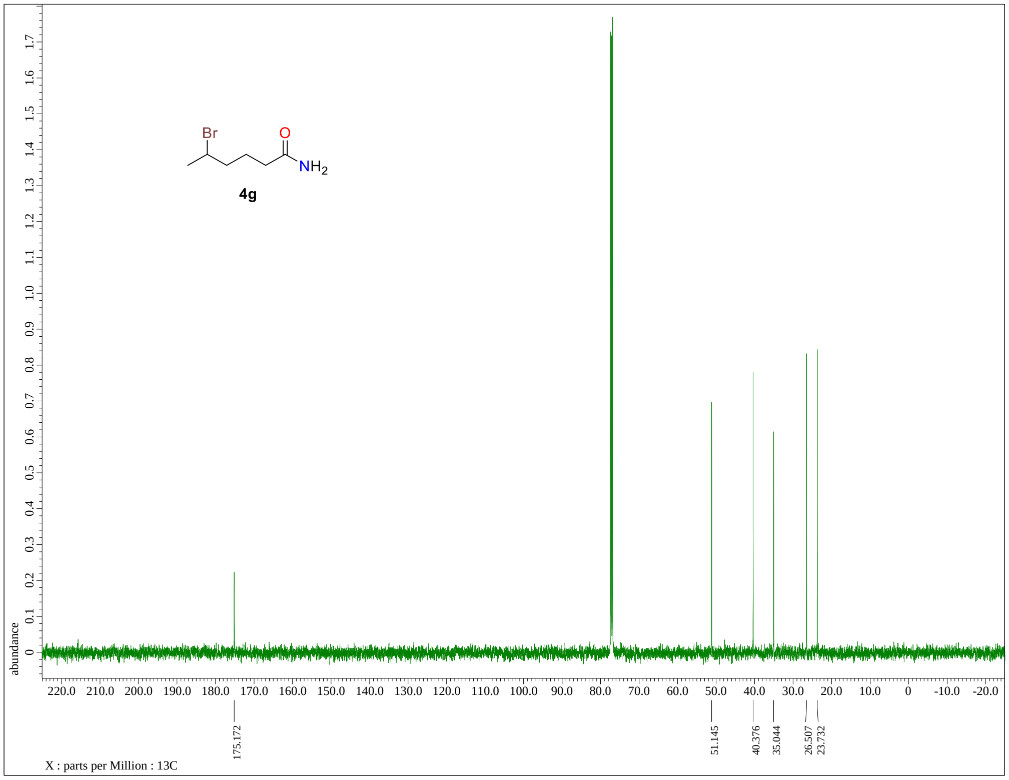

Figure S6-2. ^{13}C NMR spectrum of compound **4g** (125 MHz, $CDCl_3$)

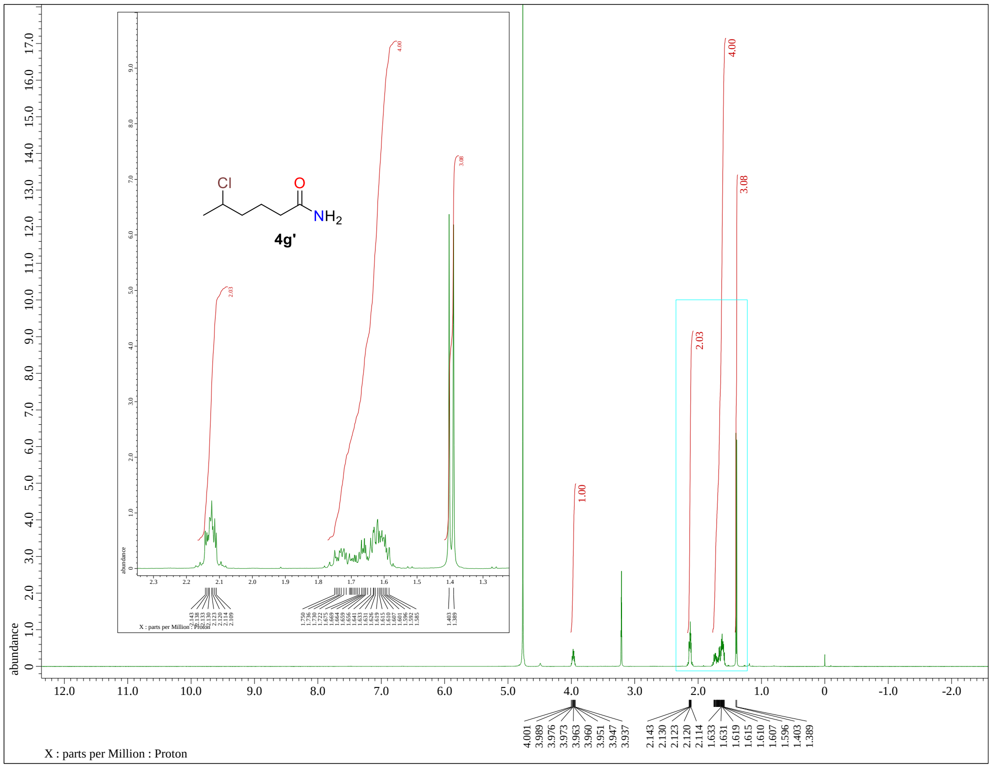

Figure S7-1. ^{1}H NMR spectrum of compound **4g’** (500 MHz, CD_3OD)

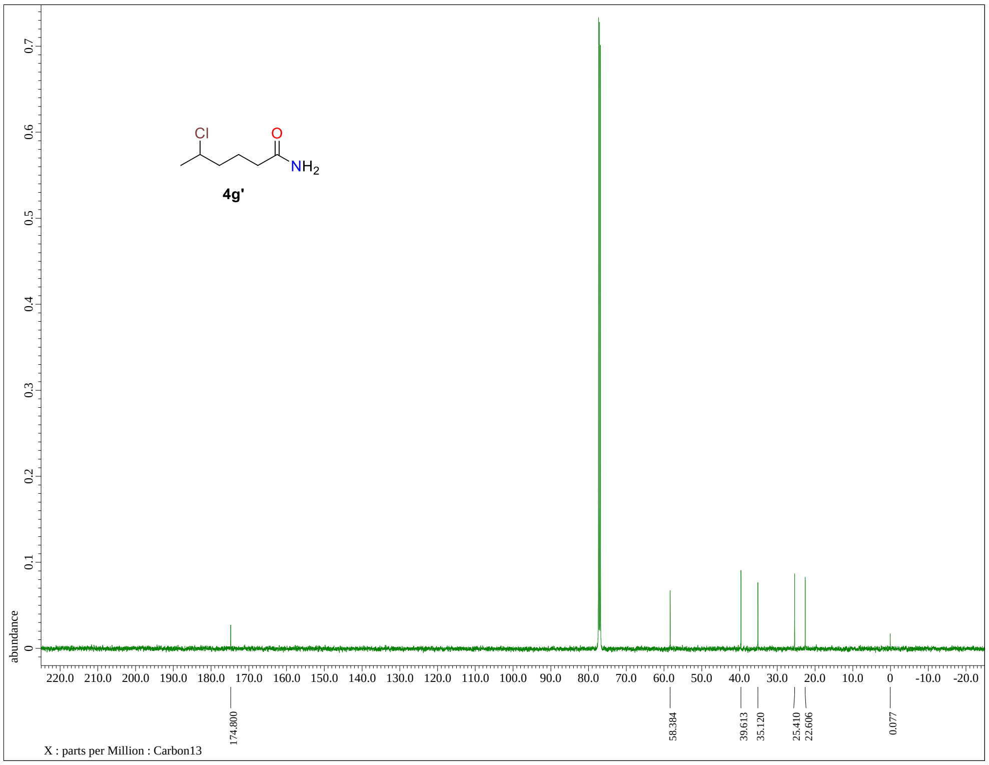

Figure S7-2. ^{13}C NMR spectrum of compound **4g’** (125 MHz, $CDCl_3$)

2. NMR Spectra of Products

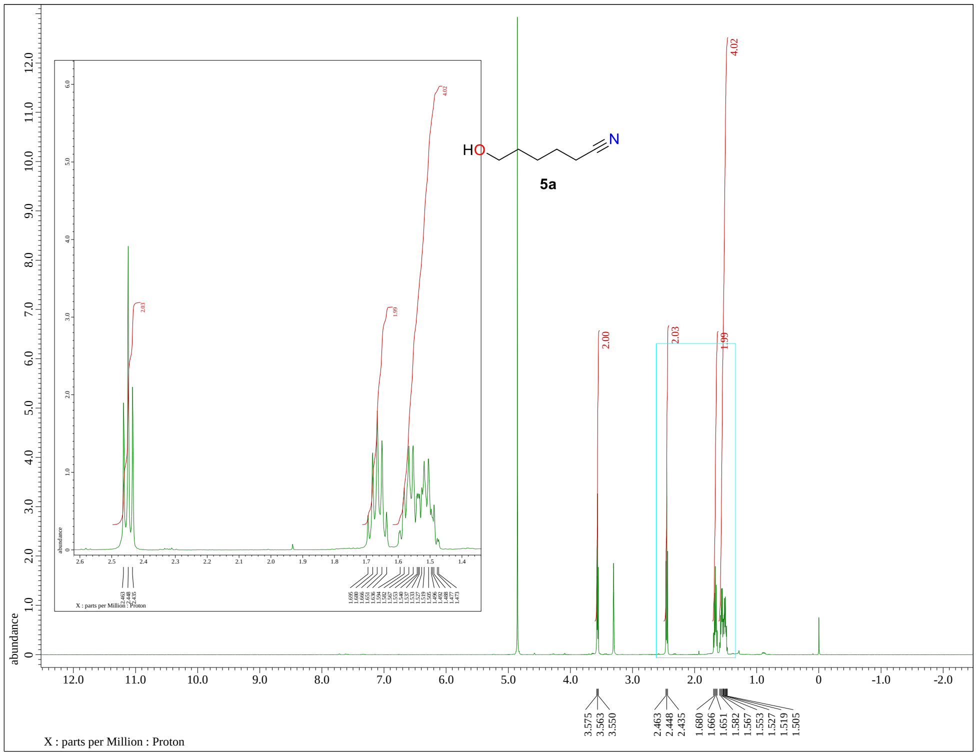

Figure S8-1. ^{1}H NMR spectrum of compound **5a** (500 MHz, CD_3OD)

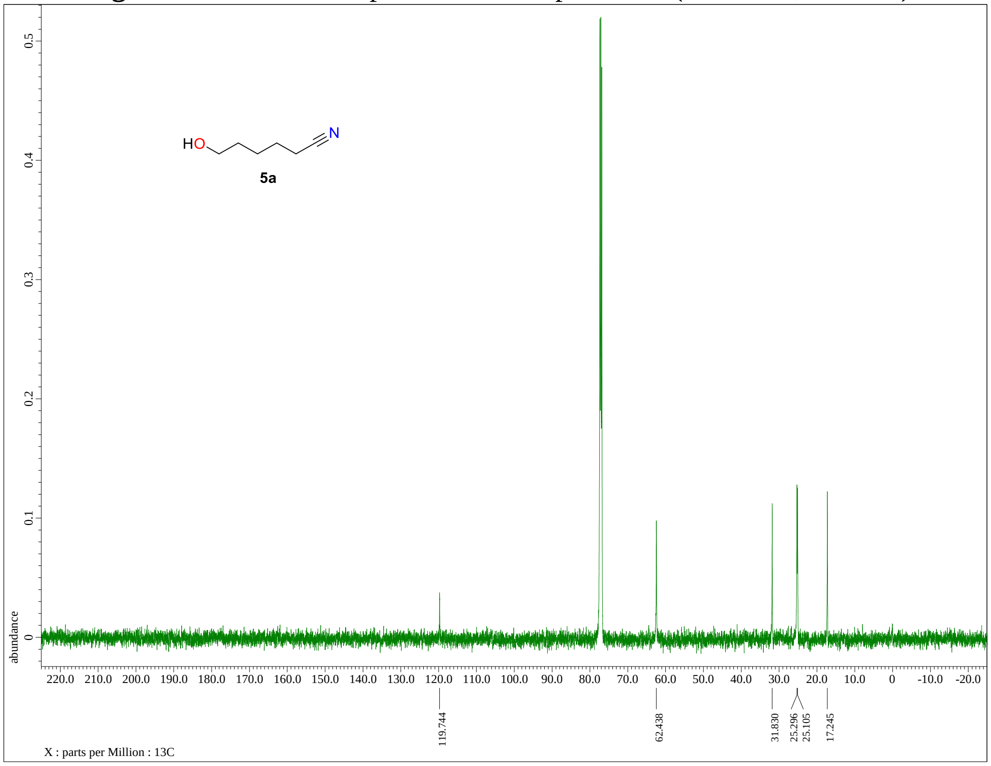

Figure S8-2. ^{13}C NMR spectrum of compound **5a** (125 MHz, $CDCl_3$)

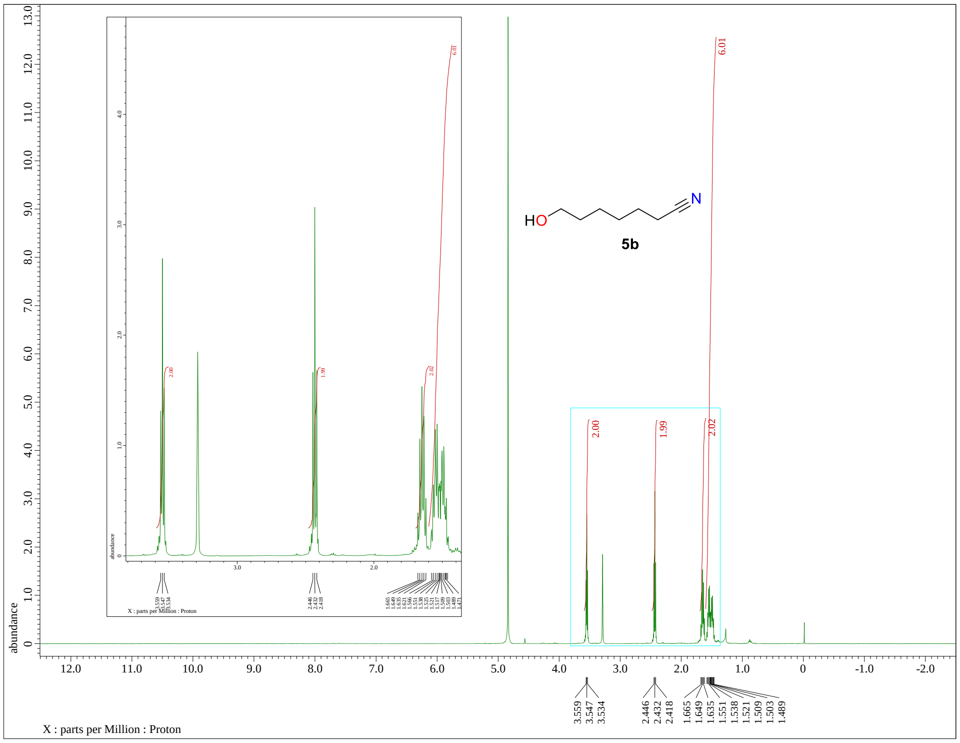

Figure S9-1. ^{1}H NMR spectrum of compound **5b** (500 MHz, CD_3OD)

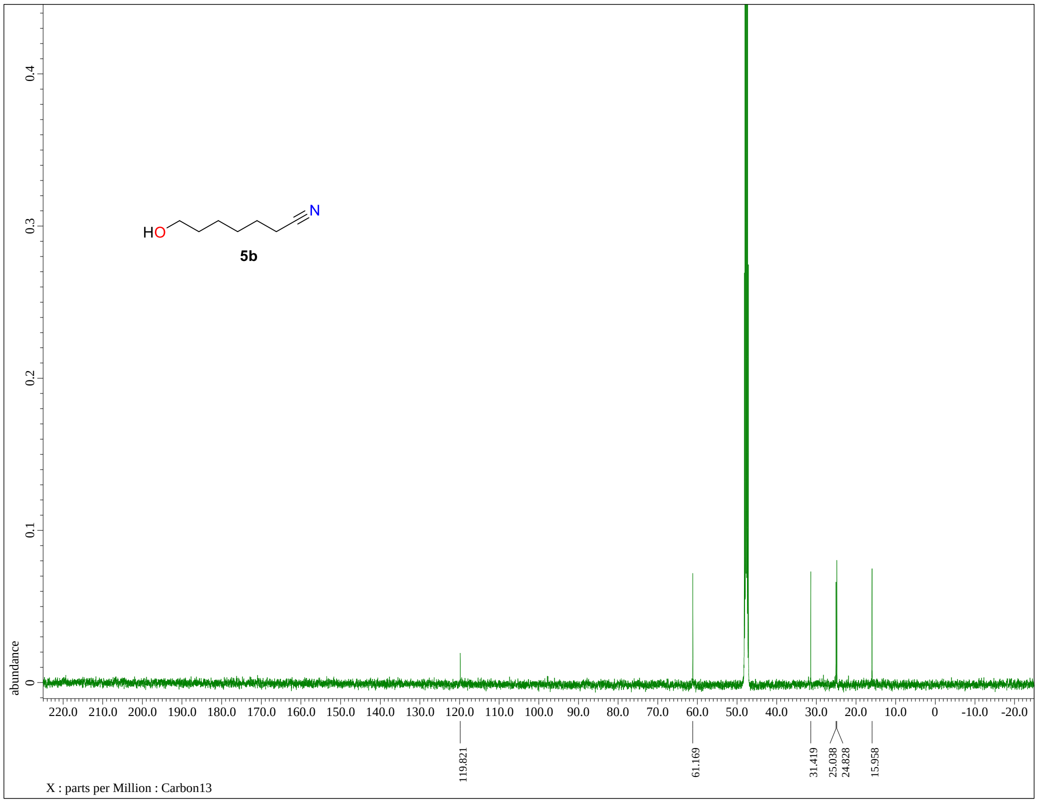

Figure S9-2. ^{13}C NMR spectrum of compound **5b** (125 MHz, CD_3OD)

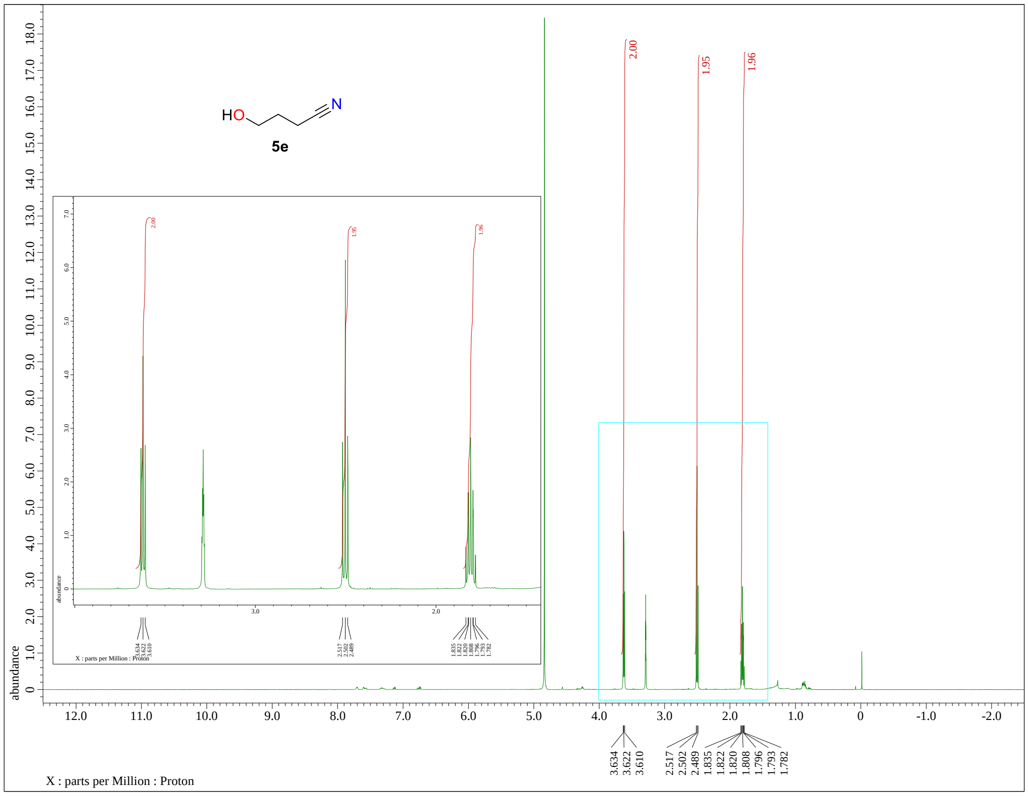

Figure S10-1. ^{1}H NMR spectrum of compound **5e** (500 MHz, CD_3OD)

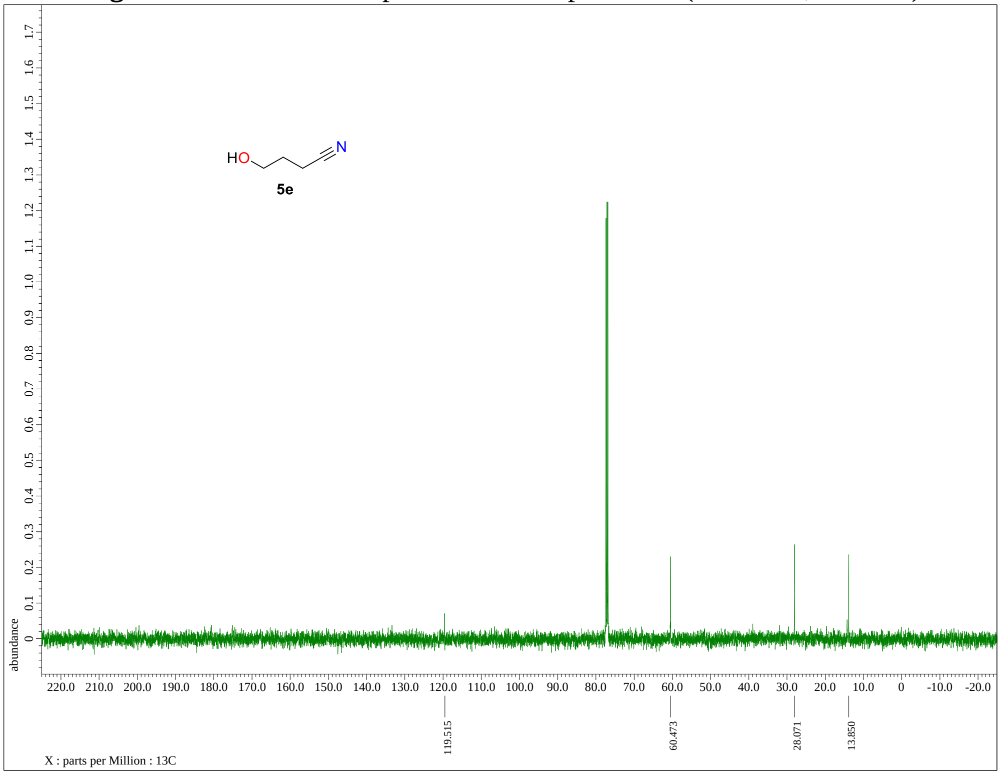

Figure S10-2. ^{13}C NMR spectrum of compound **5e** (125 MHz, $CDCl_3$)

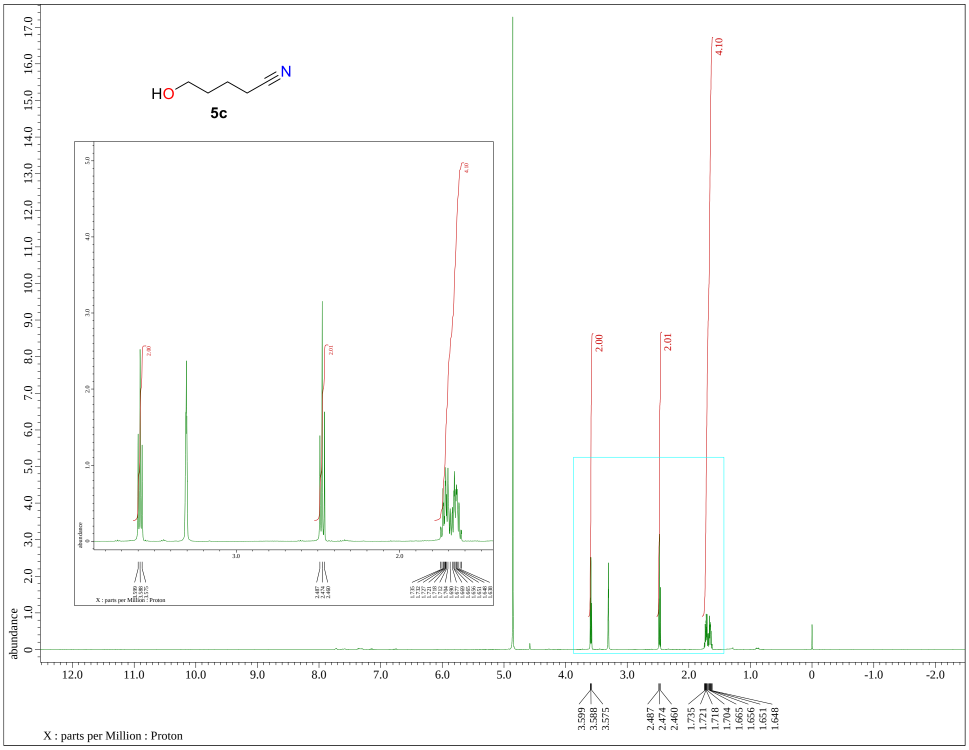

Figure S11-1. ^{1}H NMR spectrum of compound **5c** (500 MHz, CD_3OD)

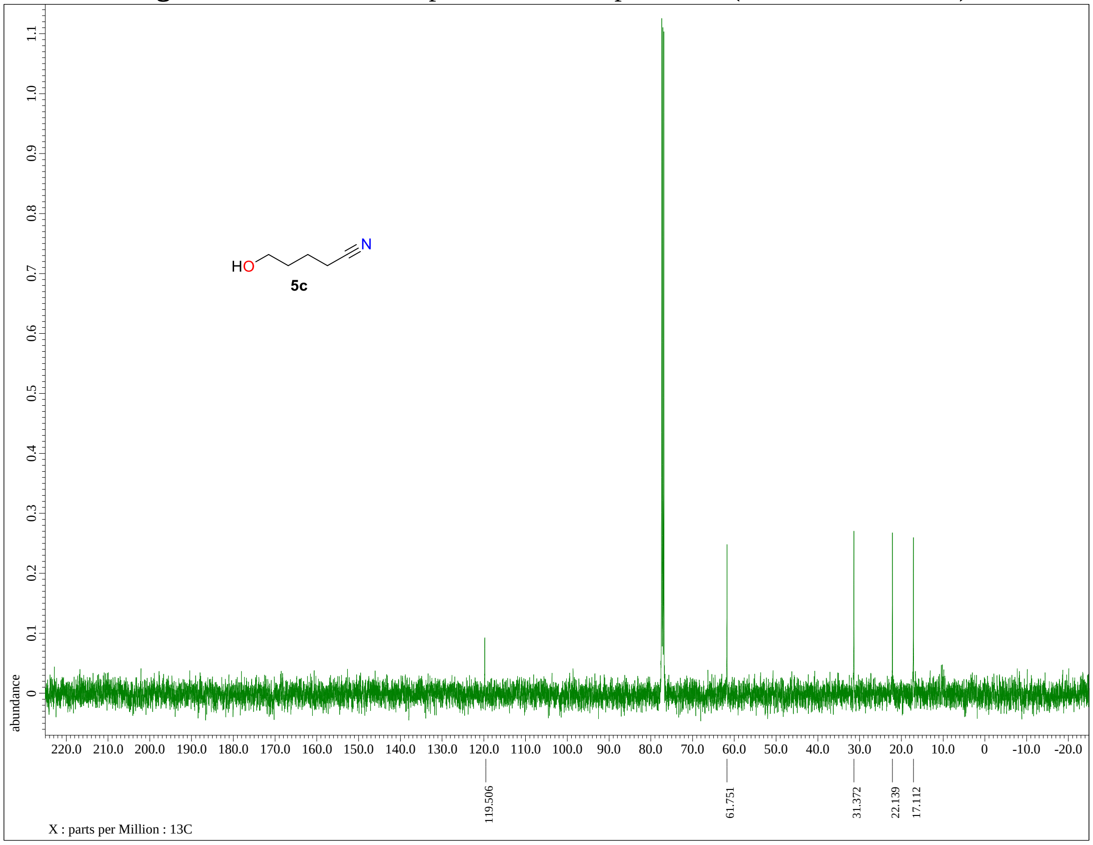

Figure S11-2. ^{13}C NMR spectrum of compound **5c** (125 MHz, $CDCl_3$)

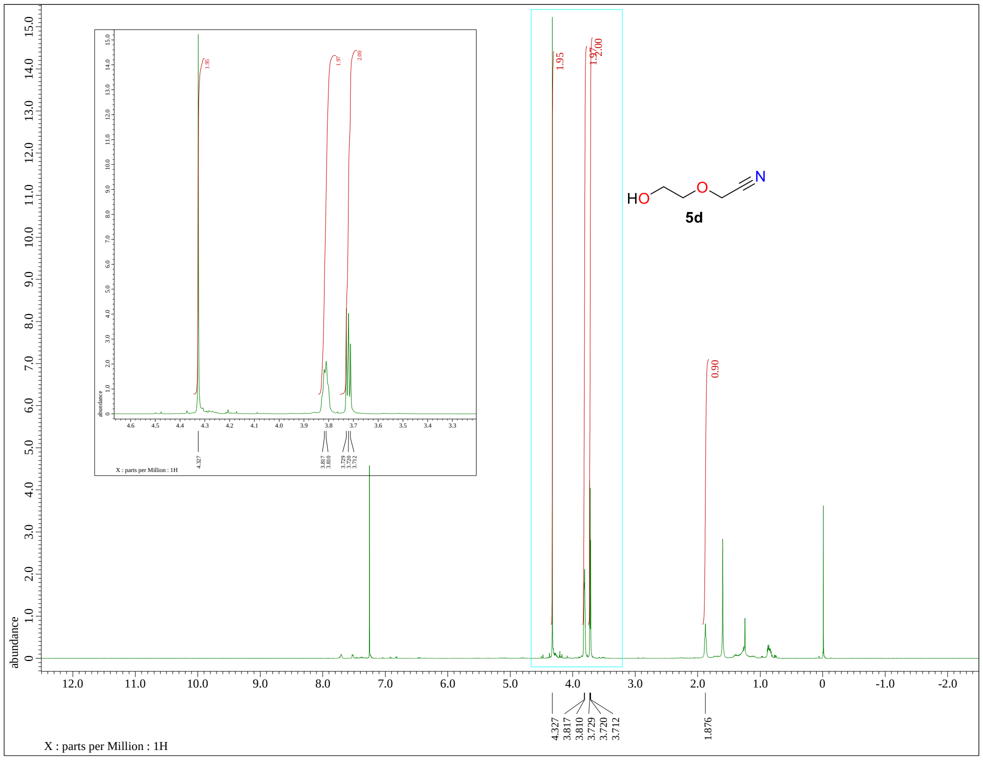

Figure S12-1. ^{1}H NMR spectrum of compound **5d** (500 MHz, $CDCl_3$)

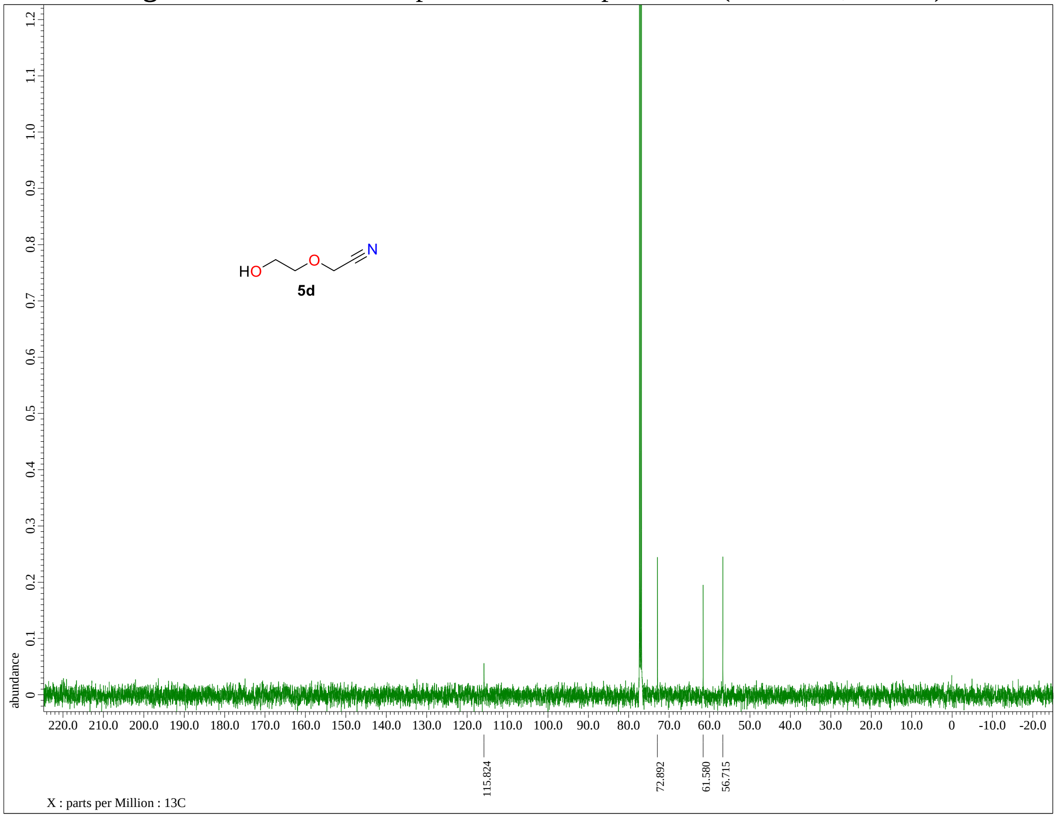

Figure S12-2. ^{13}C NMR spectrum of compound **5d** (125 MHz, $CDCl_3$)

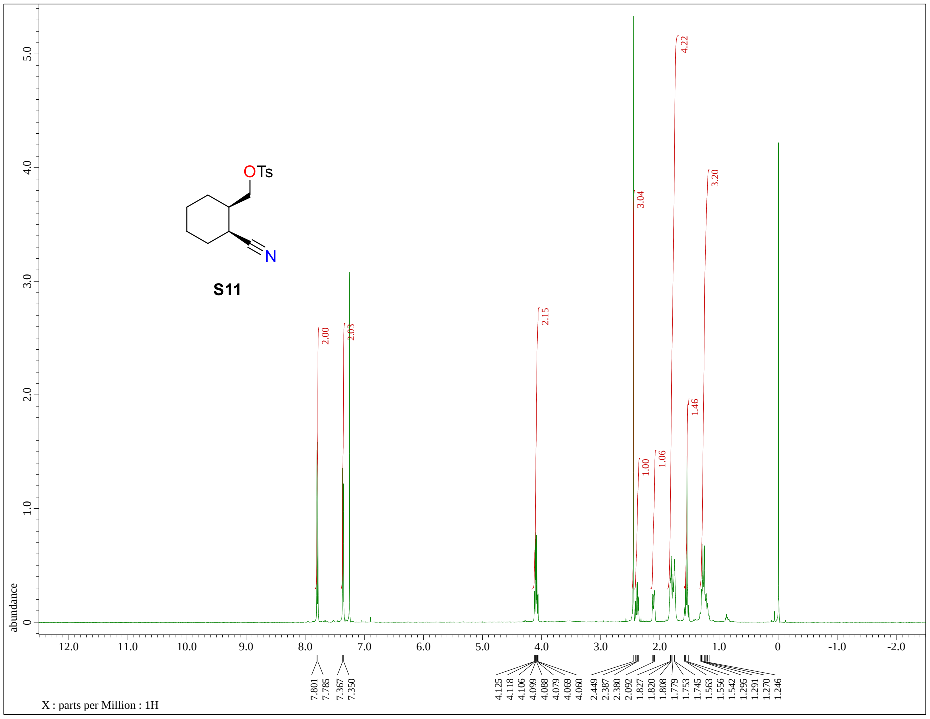

Figure S13-1. ^{1}H NMR spectrum of compound **S11** (500 MHz, $CDCl_3$)

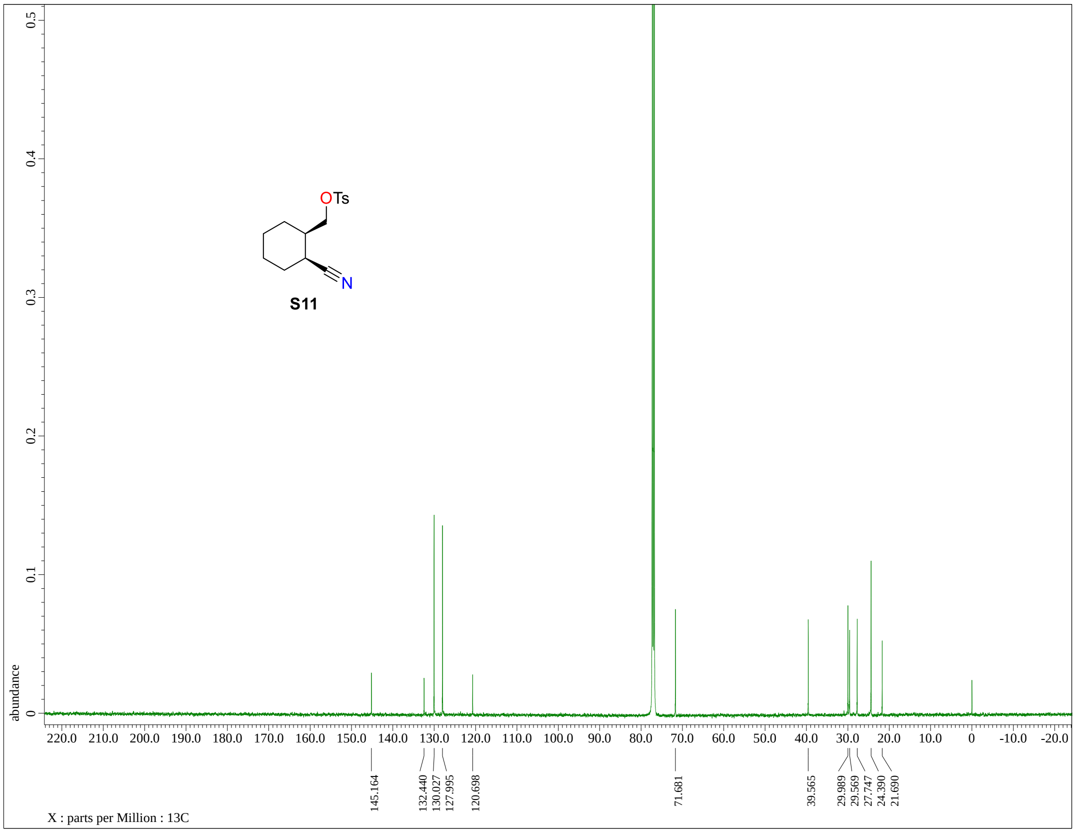

Figure S13-2. ^{13}C NMR spectrum of compound **S11** (125 MHz, $CDCl_3$)

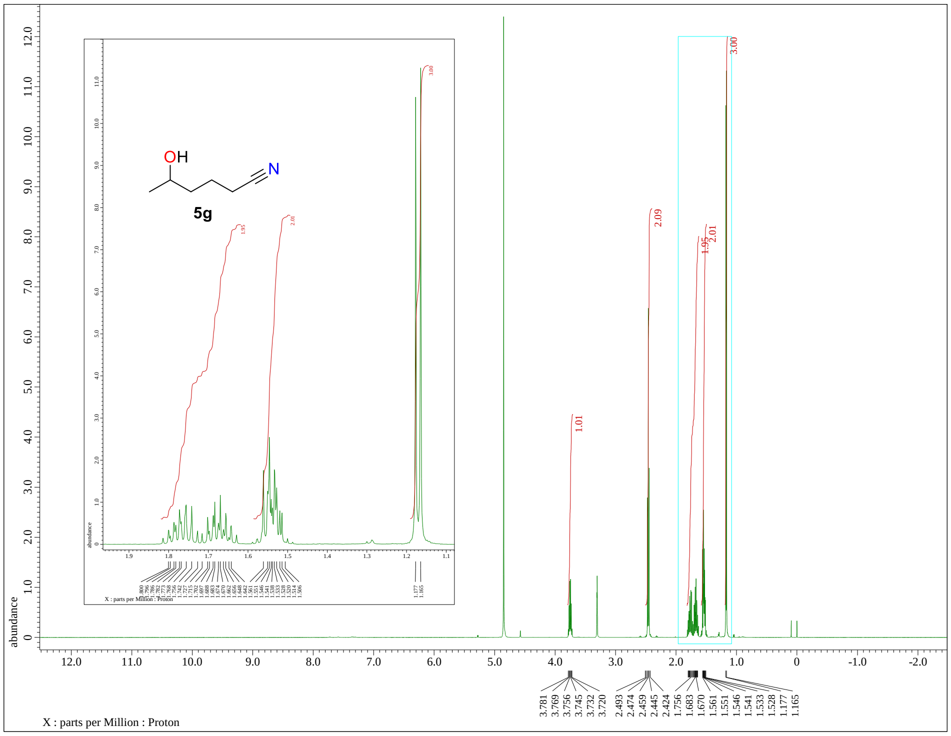

Figure S14-1. ^{1}H NMR spectrum of compound **5g** (500 MHz, CD_3OD)

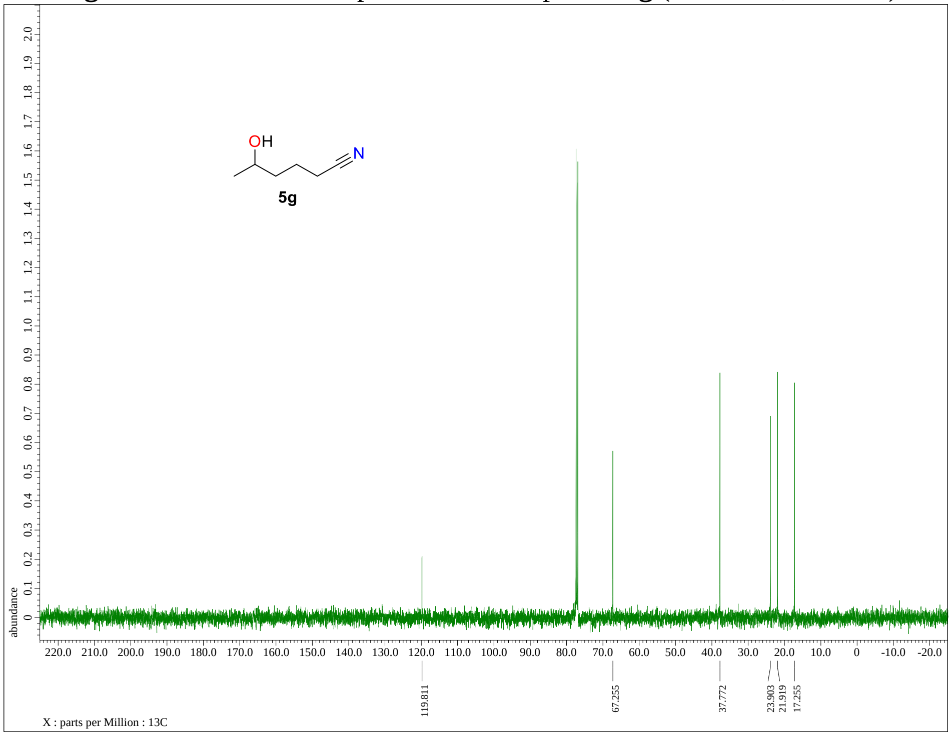

Figure S14-2. ^{13}C NMR spectrum of compound **5g** (125 MHz, $CDCl_3$)

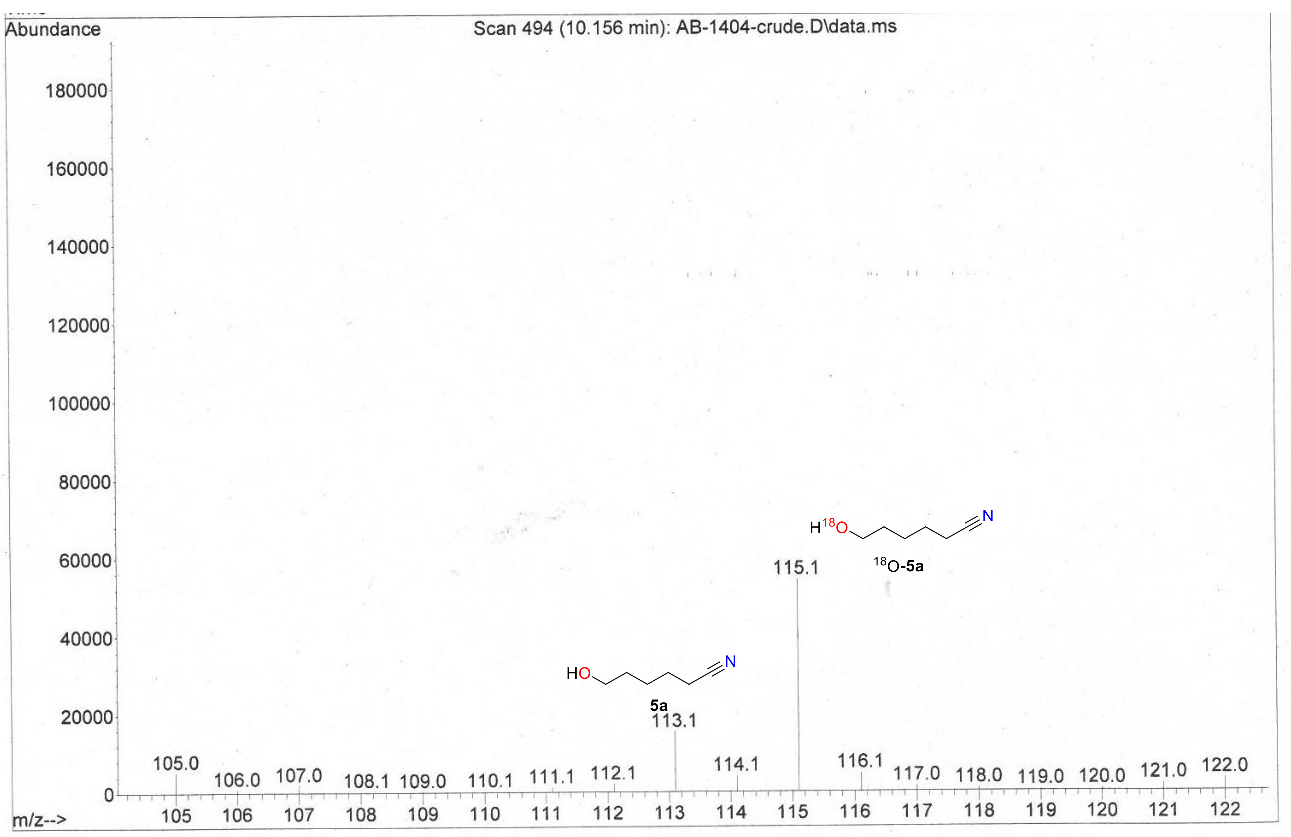

Figure S15-1. GC-MS data for **5a and** ^{18}O-**5a**
